# Characterization of phage AbpL with a terminally redundant genome and its therapeutic potential against drug-resistant *Acinetobacter baumannii* infections

**DOI:** 10.3389/fcimb.2026.1760018

**Published:** 2026-02-03

**Authors:** Defeng Liu, Kexin Zhang, Di Li, Qingqing Du, Yan Li, Jing Wang, Weiwei Jiang, Yan Qian

**Affiliations:** 1 Department of Pharmacy, The Second Affiliated Hospital of Chongqing Medical University, Chongqing, China; 2 Immunology Research Center, Medical Research Institute, Southwest University, Chongqing, China; 3 Department of Microbiology, College of Basic Medical Sciences, Army Medical University, Key Laboratory of Microbial Engineering Under the Educational Committee in Chongqing, Chongqing, China

**Keywords:** *Acinetobacter baumannii*, bacteriophage AbpL, multidrug-resistant infections, phage therapy, terminal redundancy

## Abstract

**Introduction:**

The growing global threat of multidrug-resistant *Acinetobacter baumannii* (MDR-AB) infections highlights an urgent need for novel and effective therapeutic strategies. Phage therapy has emerged as a promising alternative to conventional antibiotics. This study aimed to isolate and comprehensively characterize a novel lytic bacteriophage (phage), designated AbpL, and evaluate its therapeutic potential against MDR-AB.

**Methods:**

AbpL was isolated from sewage samples and characterized in terms of its morphology, growth characteristics, stability, and genome. Comparative genomic classification analysis was also conducted on it. *In vitro* efficacy was evaluated through time-kill assays and biofilm disruption experiments. To assess *in vivo* therapeutic potential, a murine model of lethal *A. baumannii*-induced sepsis was employed, with outcomes including survival rates, bacterial burden, serum levels of inflammatory cytokines, and histopathological evaluation.

**Results:**

AbpL is classified as a member of the genus *Friunavirus*, subfamily *Beijerinckvirinae*, family *Autoscriptoviridae*, order *Autographivirales*, and class *Caudoviricetes*. It exhibited an icosahedral head and a short non-contractile tail, and demonstrated a short latent period, a high burst size, and strong stability across a broad range of environmental conditions. AbpL showed lytic activity against 52% of clinical MDR-AB isolates and effectively disrupted pre-existing biofilms. In the murine sepsis model, a single intraperitoneal administration (multiplicity of infection = 10) conferred 100% survival, significantly reduced bacterial loads in the liver and kidneys, and attenuated systemic inflammation compared to treatment with polymyxin B. Histopathological analyses further confirmed the protective effects of AbpL and its favorable safety profile.

**Discussion:**

Owing to its strong lytic activity, environmental stability, and robust *in vitro* and *in vivo* efficacy, AbpL represents a highly promising candidate for the treatment of MDR-AB infections. Furthermore, its unique 400-bp terminal redundancy may serve as a valuable platform for future engineering via synthetic biology approaches.

## 1 Introduction

Antimicrobial resistance (AMR) has emerged as a major threat to global health in the 21st century. Recent findings indicated that approximately 4.71 million deaths worldwide were associated with bacterial AMR in 2021 (Collaborators, 2024). Without effective intervention, it is predicted that AMR related deaths may rise to 8.22 million by 2050 (Collaborators, 2024). The continuous emergence of multi-drug resistance (MDR), extensive drug resistance (XDR) and even pan-drug resistance (PDR) pathogens has led to a sharp decline in the clinical efficacy of traditional antibiotics. This decline has resulted in higher rates of treatment failure for infectious diseases, prolonged hospitalization, and a substantial increase in healthcare costs, thereby posing a serious threat to the development of modern medical systems (Macnair et al., 2024). In response to this severe situation, there is an urgent need for the global scientific community to explore and develop novel antibacterial strategies to address the challenges posed by the post-antibiotic era (Theuretzbacher, 2025).

Among the numerous drug-resistant pathogens, *Acinetobacter baumannii* has become a predominant cause of nosocomial infections, particularly in intensive care units, due to its strong environmental adaptability, persistent colonization in hospital settings, and broad drug resistance spectrum (Mea et al., 2021). This pathogen not only shows high-level resistance to commonly used antibiotics such as β-lactams, aminoglycosides, and quinolones, but also exhibits widespread production of carbapenemases and metallo-β-lactamases, resulting in a significant increase in the failure rate of carbapenem therapy (Li et al., 2025). According to the 2024 Bacterial Priority Pathogens List (BPPL) released by the World Health Organization (WHO), carbapenem-resistant *A. baumannii* was ranked as the highest priority pathogen. Therefore, it is urgent to find alternative therapeutic strategies against *A. baumannii* with enhanced drug resistance, which is also one of the important challenges faced by modern medicine (Ibrahim et al., 2021).

Bacteriophages (phages) have unique host specificity, efficient lytic ability, and self-replication characteristics, making them increasingly recognized as promising biotherapeutic agents for combating drug-resistant bacterial infections in recent years (Strathdee et al., 2023). Compared with traditional antibiotics, phages offer several advantages, including the ability to precisely target specific pathogens, thereby minimizing disruption to the normal microbiota, and overcoming established antibiotic resistance mechanisms such as efflux pumps and biofilm formation. Nevertheless, they also present limitations, such as a narrow host range and the potential for rapid development of bacterial resistance (Kulshrestha et al., 2024). At present, progress has been made in the research on *A. baumannii* phages. The NCBI Virus (https://www.ncbi.nlm.nih.gov/labs/virus/vssi/#/) has included the complete genome sequences of more than 1000 *Acinetobacter* phages, including nearly 500 A*. baumannii* phages. Several strains of *A. baumannii* phages have demonstrated *in vitro* lytic activity against clinical isolates of this pathogen. Moreover, some studies further explored the therapeutic potential of *A. baumannii* phages in murine models of lung infection or burn wound infection, showing a positive effect of reducing bacterial load and improving survival rate (Sisakhtpour et al., 2022; Zhang et al., 2022; Tu et al., 2023).

However, the practical application of *A. baumannii* phages still faces many challenges. For instance, most currently characterized phages exhibit narrow host ranges, making it difficult to cover the extensive genetic diversity of *A. baumannii* (Tan et al., 2023; Kulshrestha et al., 2024). Moreover, certain bacterial strains can evade phage infection through diverse defense mechanisms (Georjon and Bernheim, 2023; Wein et al., 2025). To address these limitations, expanding the *A. baumannii* phage resource library has become a critical step to promote phage-based therapeutic research. By isolating new *A. baumannii* phages from sewage, soil, hospital environment and other samples, combined with whole genome sequencing and comparative genomics analysis, candidate phages with broad-spectrum lytic activity or synergistic effect can be identified (Monteiro et al., 2018; Luong et al., 2020; Hatfull et al., 2022). The purpose of this study was to isolate phages capable of efficiently lysing *A. baumannii* from environmental samples, identify its biological characteristics through morphological observation, one-step growth curve determination, host spectrum analysis, multiplicity of infection (MOI) determination, and stability tests, annotate its genome function using high-throughput sequencing technology, and evaluate its potential as an antibacterial agent through *in vivo* experiments, so as to provide new insights for the development of biological strategies to prevent and control infections caused by drug-resistant *A. baumannii*.

## 2 Materials and methods

### 2.1 Bacterial strains and culture conditions

*A. baumannii* strain Ab2 was isolated from the infected burn wounds of the patients in the burn department of Southwest Hospital, Chongqing, China. Clinical isolates of multidrug resistant *A. baumannii* for host spectrum determination included Ab16716, Ab16743, Ab16812, Ab16819, Ab16835, Ab16868, Ab16886, Ab16891, Ab16900, Ab17004, Ab17006, Ab17008, Ab17023, Ab17035, Ab17111, Ab17133, Ab17161, Ab17162, Ab17163, Ab17225, and Ab17291, all of which were obtained from the laboratory department of the Second Affiliated Hospital of Chongqing Medical University. Other bacterial species, including *Escherichia coli* O157:H7, *Shigella dysenteriae* G1.126, *Salmonella enterica serovar Typhi* Ty2, and *Staphylococcus aureus* Newman, were maintained in the Department of Microbiology, College of Basic Medical Sciences, Army Medical University. All bacterial strains were identified by the VITEK 2 Compact system and confirmed to be non-duplicated. The strains were stored at -80°C in a 25% glycerol solution. The bacteria were inoculated on LB (Luria-Bertani) agar medium at 37°C overnight, or cultured in LB broth at 37°C and 200 rpm.

### 2.2 Minimum inhibitory concentration determination

The logarithmic growth phase Ab2 bacterial culture was diluted to a final concentration of 1 × 10^6^ colony forming unit (CFU)/mL. Eleven sterile 1.5 mL EP tubes were arranged in a row. The first tube received 0.8 mL of sterile PBS, while each of the remaining tubes was filled with 0.5 mL of sterile PBS. Subsequently, 0.2 mL of the original antibiotic solution (polymyxin B, initial concentration: 1,280 μg/mL) was added to the first tube and thoroughly mixed. A volume of 0.5 mL from the first tube was then transferred sequentially to the second tube, mixed well, and further serially diluted by transferring 0.5 mL to each subsequent tube up to the 11th tube. After mixing, 0.5 mL was removed from the 11th tube to maintain equal final volumes across all dilution tubes. A 12th tube, containing no antibiotic, was included as a negative control. As a result, the final drug concentrations in the series were 256, 128, 64, 32, 16, 8, 4, 2, 1, 0.5, and 0.25 μg/mL, respectively. Each antibiotic concentration was mixed with an equal volume of bacterial suspension to achieve a final bacterial concentration of 5 × 10^5^ CFU/mL. The mixtures were added to a 96-well plate (wells A2–A10). A well containing bacterial suspension without antibiotic (well A11) served as the negative control. Each condition was tested in triplicate. The 96-well plate was incubated at 37°C for over 18 hours. The next day, the turbidity in each well was visually assessed. The minimum inhibitory concentration (MIC) was defined as the lowest antibiotic concentration that completely inhibited visible bacterial growth.

### 2.3 Phage isolation

This experiment was based on a previously published protocol (Peters et al., 2022) with minor modifications. Domestic sewage samples were collected from Shapingba District, Chongqing, China. Large particles were removed by centrifugation at 5,000 × g for 10 minutes. The supernatant was filtered through a 0.45 μm membrane to remove bacteria and retain phage particles. A single colony of *A. baumannii* Ab2 was picked and inoculated in 200 mL LB broth and incubated at 37°C with shaking at 200 rpm for 5 hours. Then, 200 mL of filtered sewage was co-cultured with 200 mL of Ab2 bacterial suspension overnight to allow phage enrichment. Following incubation, the mixture was centrifuged at 5,000 × g for 10 minutes, and the supernatant was filtered by 0.45 μm membrane (Sangon, Shanghai). Phage particles were then isolated using the double-layer agar plaque assay (Jiang et al., 2024). In brief, 10 μL of gradient-diluted supernatant was added to 200 μL of logarithmic-phase host bacteria. After incubation at room temperature for 10 minutes, 3 mL of pre-warmed semi-solid LB agar medium (containing 0.4% agarose, approximately 50°C) was added. The mixture was vortexed briefly to ensure homogeneity and immediately poured onto an LB agar medium (containing 1.5% agar). After allowing the top layer to solidify at room temperature for approximately 15 minutes, the plates were inverted and incubated overnight at 37°C. The formation of phage plaques was examined the following day. A single plaque was selected and subjected to three rounds of single-plaque isolation and propagation to ensure phage homogeneity. For phage propagation, a single plaque was selected and inoculated into fresh Ab2 bacterial culture, and incubated at 37°C with shaking at 200 rpm for approximately 5 hours until the bacterial suspension became visibly clear. After centrifuging the phage lysate at 5000 × g for 10 minutes, the phage supernatant was collected and stored at 4°C for standby. The double-layer agar plate method was used to calculate the titer of the phage preparation.

### 2.4 Phage purification

The phage was purified according to the previously reported method (Tang et al., 2018). Briefly, *A. baumannii* Ab2 was inoculated in 300 mL LB broth, and then incubated at 37°C and 200 rpm until reaching the early logarithmic phase. Then, 300 μL of phage preparation with a titer of ~1 × 10^10^ PFU (plaque forming unit)/mL was added and co-cultured for about 5 hours until bacterial lysis. DNase I (5 μg/mL) and RNase A (10 μg/mL) were added to the lysate to the final concentration of 1 μg/mL, followed by incubation at 37°C for 30 minutes, then NaCl was added to a final concentration of 5.84 g/100 mL of the lysate, and centrifuged at 10,000 × g for 10 minutes after ice bath for 1 hour. The supernatant was collected, and polyethylene glycol 8000 (PEG 8000) was added to a final concentration of 10% (w/v). After overnight incubation on ice, the sample was centrifuged at 12,000 × g for 10 minutes. The precipitates were collected and resuspended with 2~3 mL TM buffer (pH 7.5, containing 0.2% MgSO_4_·7H_2_O). Following two rounds of chloroform extraction with equal volumes, the upper aqueous phase was collected to obtain crude phage particles. Cesium chloride solutions with densities of 1.3 g/mL, 1.5 g/mL, and 1.7 g/mL were prepared in TM buffer, and the solutions were layered into the ultracentrifugation tube in descending order of density. The crude phage suspension was carefully loaded onto the top of the gradient, and the tube was balanced with additional TM buffer before ultracentrifugation at 20,000 × g at 4°C for 2 hours. The visible phage band was extracted using a syringe, and excess cesium chloride was removed by dialysis.

### 2.5 Transmission electron microscopy

The purified phage preparation (~1 × 10^10^ PFU/mL) was observed under a high-resolution electron microscope (Tang et al., 2018). The procedure was as follows: 20 µL of the purified phage suspension was placed onto a carbon-coated copper grid and allowed to adsorb at room temperature for 15 minutes. The sample was then negatively stained with 2% phosphotungstic acid for 10 seconds and air-dried. Observation was performed using a JEM-1400 Plus transmission electron microscope (JEOL, Japan) at an accelerating voltage of 80 kV and a magnification of 100,000 ×.

### 2.6 Determine the optimal multiplicity of infection of the phage

The logarithmic growth phase of *A. baumannii* Ab2 (~10^8^ CFU/mL) was mixed with the phage preparation (~1 × 10^10^ PFU/mL) according to the multiplicity of infection (MOI) gradient (100, 10, 1, 0.1, 0.01, 0.001), incubated for 3 minutes, and then centrifuged at 10,000 × g for 3 minutes. The supernatant was discarded, and the pellet was washed twice with PBS buffer before being resuspended in 3 mL of LB broth. After incubation with shaking at 37°C and 200 rpm for approximately 5 hours until the bacterial suspension became visibly clear, the lysate was centrifuged to remove bacterial debris. The phage titer in the supernatant was determined using the double-layer agar plaque assay (Le et al., 2024). The MOI corresponding to the highest phage titer was selected as the optimal MOI. Three parallel experiments were set to ensure the reliability of the results.

### 2.7 Determination of one-step growth curve

The one-step growth characteristics of phages were identified according to the previously reported method (Li et al., 2024). A 3 mL aliquot of logarithmic-phase *A. baumannii* Ab2 culture (~10^8^ CFU/mL) was mixed with an excess of phage preparation (~1 × 10^10^ PFU/mL), and unbound phages were removed by centrifugation after adsorption at room temperature for 3 minutes. The pellet was resuspended in LB broth and cultured at 37°C. Starting from 0 min, 100 μL samples were collected every 5 min. Each sample was centrifuged, and the supernatant was subjected to serial dilution for phage titer determination using the double-layer agar method. Monitoring continued until 120 min or until complete bacterial lysis was observed. Three parallel replicates were performed to ensure data reliability. Finally, the one-step growth curve of the phage was plotted with time (in minutes) on the abscissa and phage titer on the ordinate.

### 2.8 Phages stability testing

The stability of phage AbpL was evaluated using a previously reported method (Peters et al., 2023; Wang et al., 2024a). Briefly, the purified phage preparation (~1 × 10^10^ PFU/mL) was treated at 4°C, 37°C, 55°C, 70°C, and 80°C for 1 hour, respectively. After gradient dilution, the phage titer was determined by dot assay to assess its thermal tolerance. To evaluate pH stability, the phage was mixed with buffers of pH ranging from 1 to 12 and incubated at 37°C for 1 hour. The phage titer was then measured, with LB broth serving as the control, to determine the acid-base tolerance range. For chloroform sensitivity testing, the phage supernatant was gently mixed with chloroform for 2 minutes and centrifuged at 5000 × g for 10 minutes, after which the titer of the phage in the supernatant was determined. Additionally, the phage suspension was exposed to ultraviolet light (the radiation intensity of the ultraviolet lamp is approximately 80 μW/cm^2^), and samples were collected every 10 minutes over a 1-hour period. After gradient dilution, the phage survival rate was measured to evaluate the ultraviolet inactivation effect. All experiments were performed in triplicate.

### 2.9 Host range determination

Bacterial strains used for host range determination were listed in **Supplementary Table S1**. Bacterial solution of 30 μL was added to 3 mL of fresh LB broth, and cultured at 37°C with shaking at 200 rpm for about 3 hours to the early logarithmic growth (OD_600_ ≈ 0.0625~0.125, no more than 0.125). Approximately 200 μL of host bacteria in the logarithmic growth phase was mixed with semi-solid LB agar medium at approximately 50°C, vortexed thoroughly, and overlaid onto a pre-solidified LB agar plate to form a double-layer agar system. The phage preparation (~1 × 10^10^ PFU/mL, 10 μL) was then spotted onto the surface of the bacterial soft lawn. An equal volume of LB medium without phage served as the negative control. The plate was incubated at 37°C overnight, and the phage lysis activity was observed the following day. Lysis was indicated by the presence of a clear zone (+), while no lysis was marked as “−”. The experiment was repeated three times independently.

### 2.10 Extraction and purification of phage DNA

The phage nucleic acid was extracted and purified by referring to the previously reported method (Lu et al., 2013). In brief, DNase I and RNase A were added to the purified phage preparation (~1 × 10^10^ PFU/mL) to achieve final concentrations of 5 μg/mL and 1 μg/mL, respectively. Then the mixture was incubated at 37°C for 1 hour to degrade any residual host nucleic acids. Subsequently, ethylenediamine tetraacetic acid (EDTA) (final concentration: 20 mmol/L) was added to inactivate DNase I, followed by the addition of proteinase K (50 μg/mL) and SDS (0.5%) to incubate at 56°C for 1 hour, which facilitated the release of phage genomic DNA and degradation of residual enzymes. The lysate was sequentially extracted with equal volumes of equilibrated phenol and chloroform, each centrifuged at 5,000 × g for 10 minutes. After collecting the upper aqueous phase, two volumes of absolute ethanol were added, and the mixture was incubated at –20°C for 1 hour or overnight to precipitate the DNA. Following centrifugation at 12,000 × g for 20 minutes, the DNA pellet was collected, washed with 70% ethanol, air-dried, and finally dissolved in tris EDTA (TE) buffer. The extracted nucleic acid was quantified, aliquoted, and stored at –20°C for further use.

### 2.11 Genome sequencing and annotation

The purified DNA of phage AbpL was submitted to the Chongqing Branch of Beijing Qingke Biotechnology Co., Ltd. for sequencing. Genome amplification and deep sequencing were performed using Illumina sequencing technology, and an Illumina paired-end (PE) library was constructed. Read data were processed for quality control and adapter trimming with fastp (https://github.com/OpenGene/fastp). The raw sequencing data were assembled using SPAdes v3.5.0 (Bankevich et al., 2012), resulting in the complete genome sequence of phage AbpL. The AbpL genome was initially annotated using the RAST server (McNair et al., 2018), and the annotation results were further validated using GeneMark (Brůna et al., 2024) and BLAST (Singh and Raghava, 2016; Camacho et al., 2023). tRNA genes within the AbpL genome were predicted using tRNAscan-SE 2.0 (Chan et al., 2021). Potential virulence factors were identified by comparing the genome sequence against the Virulence Factor Database (VFDB) (Liu et al., 2022). Antibiotic resistance genes were predicted using ResFinder 4.6.0 (Bortolaia et al., 2020). The lifestyle (lytic or lysogenic) of phage AbpL was predicted using DeePhage (Wu et al., 2021). Genome visualization was achieved using Proksee (Grant et al., 2023) and the Blast Ring Image Generator (BRIG) (Alikhan et al., 2011). Finally, the annotated AbpL genome sequence was submitted to GenBank (https://www.ncbi.nlm.nih.gov/genbank/) via BankIt (https://www.ncbi.nlm.nih.gov/WebSub/).

### 2.12 Genome termini analysis

The terminal sequence of the phage AbpL genome was identified using a terminal run-off sequencing method (Lu et al., 2013). The genomic sequence of phage AbpL was analyzed by in silico restriction enzyme digestion using the GeneQuest module of the DNASTAR Lasergene software (https://www.dnastar.com/software/lasergene/). The genomic DNA of AbpL was digested with restriction endonucleases NdeI and HpaI (Thermo Fisher Scientific, USA), and the fragments were analyzed by agarose gel electrophoresis. The 5’ and 3’ terminal fragments of the phage AbpL genomic DNA were recovered using a DNA Gel Recovery Kit (Vazyme, China). Sequencing primers were designed for both ends: P5’ (5’–CCTTTCTATACGTTGAT–3’) for the 5’ end and P3’ (5’–CTGTGTACTTATCTACAGA–3’) for the 3’ end. Sequencing was performed by the Beijing Genomics Institute (Beijing, China). The resulting sequences were aligned with the AbpL genome sequence to determine the length and composition of the terminal repeats. MEGA12 (https://www.megasoftware.net/) was employed to analyze the phylogenetic relationships of the terminal sequence within the AbpL phage genome and other related sequences.

### 2.13 Structural protein analysis

Phage structural protein analysis was performed according to the previously reported methods (Lu et al., 2013; Tang et al., 2018). Briefly, 100 μL of purified phage preparation (~1 × 10^10^ PFU/mL) was mixed with loading buffer, denatured at 100°C, and subjected to sodium dodecyl sulfonate-polyacrylamide gel electrophoresis (SDS-PAGE) under the following conditions: initial voltage of 90 V for 20 minutes, followed by 130 V for 30 minutes. After electrophoresis, the gel was removed, stained with Coomassie Brilliant Blue for 2 hours, and subsequently destained until the background became transparent. Protein band molecular weights were determined by comparison with a protein molecular weight marker. Target protein bands were excised, de-stained using acetonitrile/NH_4_HCO_3_, and subjected to protein reduction, alkylation, trypsin digestion, and peptide desalting. The resulting peptides were analyzed by mass spectrometry to obtain peptide mass data. Protein identification and functional annotation were conducted by searching against the phage AbpL genome annotation database. Data reliability was assessed by analyzing the distribution of precursor and fragment ion mass tolerances. Finally, the electrophoretic bands were correlated with the mass spectrometry identification results to determine the molecular weight and functional annotation of the phage structural proteins.

### 2.14 Comparative genomics analysis

The genomic sequences of 50 phages with a maximum score greater than 15,900 when compared to AbpL were selected for phylogenetic analysis. The VICTOR online tool (https://ggdc.dsmz.de/victor.php) (Meier-Kolthoff and Göker, 2017) was used to perform the analysis and construct the phylogenetic tree. The proteomic phylogenetic tree of phage AbpL and other dsDNA phages was constructed using the ViPTree server (version 4.0) (Nishimura et al., 2017a, b). A total of 5,633 dsDNA phage genomes (**Supplementary Table S2**), including that of AbpL, were included in the analysis. This analysis was performed using the default parameters provided by the server. Taxonomic classification of phage AbpL and the calculation of average nucleotide identity (ANI) were performed using taxMyPhage (https://ptax.ku.dk/) (Millard et al., 2025). Additionally, EasyFig (http://mjsull.github.io/Easyfig/) (Sullivan et al., 2011) was employed to select the 4 phages most closely related to AbpL. A tBlastX linear comparison analysis was conducted between these phages and AbpL, and the results were visualized through graphical representation. BRIG (BLAST Ring Image Generator, https://sourceforge.net/projects/brig/) (Alikhan et al., 2011) comparison of complete genome sequences of AbpL with other 13 most related phages was performed with default parameters.

### 2.15 *In vitro* time-kill curve of AbpL

The method was adapted from a previously reported protocol with minor modifications (Li et al., 2024). The host strain Ab2 was cultured to the early logarithmic growth phase (approximately 2 hours, OD_600_ = 0.1–0.2). A volume of 180 μL of bacterial suspension was added to each well of a 96-well plate, followed by the addition of 20 μL of phage preparation in descending order of phage titer (ranging from 1 × 10^9^ PFU/mL to 1 × 10^1^ PFU/mL). Polymyxin B with a final concentration of 1 MIC (8 μg/mL) was added to the control group, while 20 μL of PBS was added to the blank group. After sample addition, the 96-well plate was placed in a microplate reader and incubated for 12 hours, during which the OD_570_ value of each well was recorded. Each experimental group was set up in triplicate.

### 2.16 Antibiofilm activity test

This experiment was conducted following a previously reported method (Lu et al., 2021) with minor modifications. The host strain Ab2 was cultured to the logarithmic growth phase under standard aerobic conditions at 37°C with shaking. After centrifugation at 5,000 × g for 10 minutes, the bacterial pellet was resuspended and diluted to a concentration of 1 × 10^8^ CFU/mL in LB broth supplemented with 1% glucose. A volume of 200 μL of the bacterial suspension was added to each well of a 96-well plate, with LB broth alone serving as the negative control. The plate was incubated at 27°C for 24 hours. The supernatant was gently removed, and 200 μL of polymyxin B at a final concentration of 2 ~ 32 μg/mL (1/4 MIC, 1/2 MIC, 1 MIC, 2 MIC, 3 MIC, and 4 MIC) or a phage preparation gradient solution with titers ranging from 1 × 10^1^ to 1 × 10^8^ PFU/mL was added to the respective wells. Untreated bacterial biofilm served as the positive control. Each group was set up in triplicate and incubated for an additional 24 hours. Following incubation, the supernatant was removed, and the wells were washed three times with sterile PBS and allowed to air dry. Subsequently, 200 μL of 0.1% crystal violet was added to each well and incubated for 15 minutes. The excess dye was discarded, and the wells were washed twice with distilled water and air-dried again. Finally, 200 μL of 95% ethanol was added to dissolve the stained biofilm for 30 minutes, and the optical density (OD) was measured at 570 nm. Biofilm inhibition was calculated using the following formula: Biofilm (%) = [(OD_570_ of treated sample – OD_570_ of blank)/(OD_570_ of untreated – OD_570_ of blank)] × 100%.

### 2.17 Construction of murine peritoneal infection model

Twenty-five male BALB/c mice aged approximately 7 weeks were selected and randomly divided into five groups. The mice were acclimatized with free access to normal water and rodent chow for three days without any intervention. On the fourth day, all mice were intraperitoneally injected with bacterial suspensions. The groups were designated as the control group, experimental group A, experimental group B, experimental group C, and experimental group D. Experimental group A received an intraperitoneal injection of 100 μL of Ab2 at a concentration of 1 × 10^6^ CFU/mL, group B received 1 × 10^7^ CFU/mL, group C received 5 × 10^7^ CFU/mL, and group D received 1 × 10^8^ CFU/mL. The control group received an intraperitoneal injection of 100 μL of sterile PBS. All bacterial cultures were centrifuged and resuspended in sterile PBS prior to injection. The day of injection was designated as day 0. Daily observations were conducted to record survival status, feeding behavior, fur condition, and general health status. A survival curve was generated based on continuous observation over 7 days. At the end of the experiment, mice were anesthetized via isoflurane inhalation (induction at 4% for 20 seconds, followed by maintenance at 2%) prior to euthanasia by cervical dislocation.

### 2.18 Phage therapy and therapeutic effect evaluation

This experiment was conducted following previously reported methods (Lu et al., 2021; Li et al., 2024). An acute abdominal infection model was established by intraperitoneal injection of 100 μL of Ab2 bacterial suspension (approximately 1 × 10^8^ CFU/mL) into fifteen mice. The mice were randomly assigned to five groups (n = 5 per group): a negative control group (injected with 100 μL of PBS) and three experimental groups (A, B, and C), which received intraperitoneal injections of 1, 5, or 10 mg/kg polymyxin B (PB), respectively. Treatment was initiated 2 hours post-infection. Over the following 7 consecutive days, survival rates, activity levels, fur condition, and body weight changes were monitored to preliminarily determine the effective dose range of polymyxin B. In a subsequent experiment, thirty Ab2-infected mice were randomly divided into five groups (n = 6 per group): a negative control group (PBS), a positive control group (10 mg/kg PB), and three experimental phage groups receiving a single intraperitoneal injection of 100 μL purified phage preparation with titers of 1 × 10^7^ PFU/mL, 1 × 10^8^ PFU/mL, 1 × 10^9^ PFU/mL, respectively, with corresponding MOIs of 0.1, 1, and 10. All treatments were administered 2 hours after infection. The 7-day survival rate and clinical symptoms were continuously recorded.

On the seventh day post-infection, the mice were anesthetized via isoflurane inhalation (induction at 4% for 20 seconds, followed by maintenance at 2%) prior to euthanasia by cervical dislocation. The kidneys and livers were aseptically collected, and 1 mL of PBS was added to each tissue for homogenization. Following serial dilution of the homogenates, bacterial loads were quantified using the plate counting method, and phage titers were determined by the double-layer agar plaque assay to evaluate phage colonization and clearance efficiency in the organs. In a separate experiment, mice were euthanized 7 hours after treatment, and liver and kidney tissues were fixed in 4% paraformaldehyde for 24 hours. After paraffin embedding, tissue sections (4 μm thickness) were prepared and stained with hematoxylin and eosin (H&E) to assess inflammatory infiltration, tissue necrosis, and other histopathological changes. Additionally, 7 hours after treatment, blood was collected from the retro-orbital plexus of infected mice following removal of the whiskers to prevent hemolysis. Whole blood (150 ~ 300 μL) was collected from each mouse and stored overnight at 2 ~ 8°C. The next day, samples were centrifuged at 1000 × g for 10 minutes, and the supernatant serum was carefully collected. Serum levels of the inflammatory cytokines IL-6 and TNF-α were measured using the ELISA double antibody sandwich method.

### 2.19 Statistical analysis

All data were analyzed and compared using one-way ANOVA with GraphPad Prism 9.0 software. The error bars represent the standard deviation (SD), indicating the variability within the data. A p-value < 0.05 was considered statistically significant, and p-values < 0.01 or lower indicated highly significant differences.

## 3 Results

### 3.1 The biological characteristics of phage AbpL

**Figure 1:**
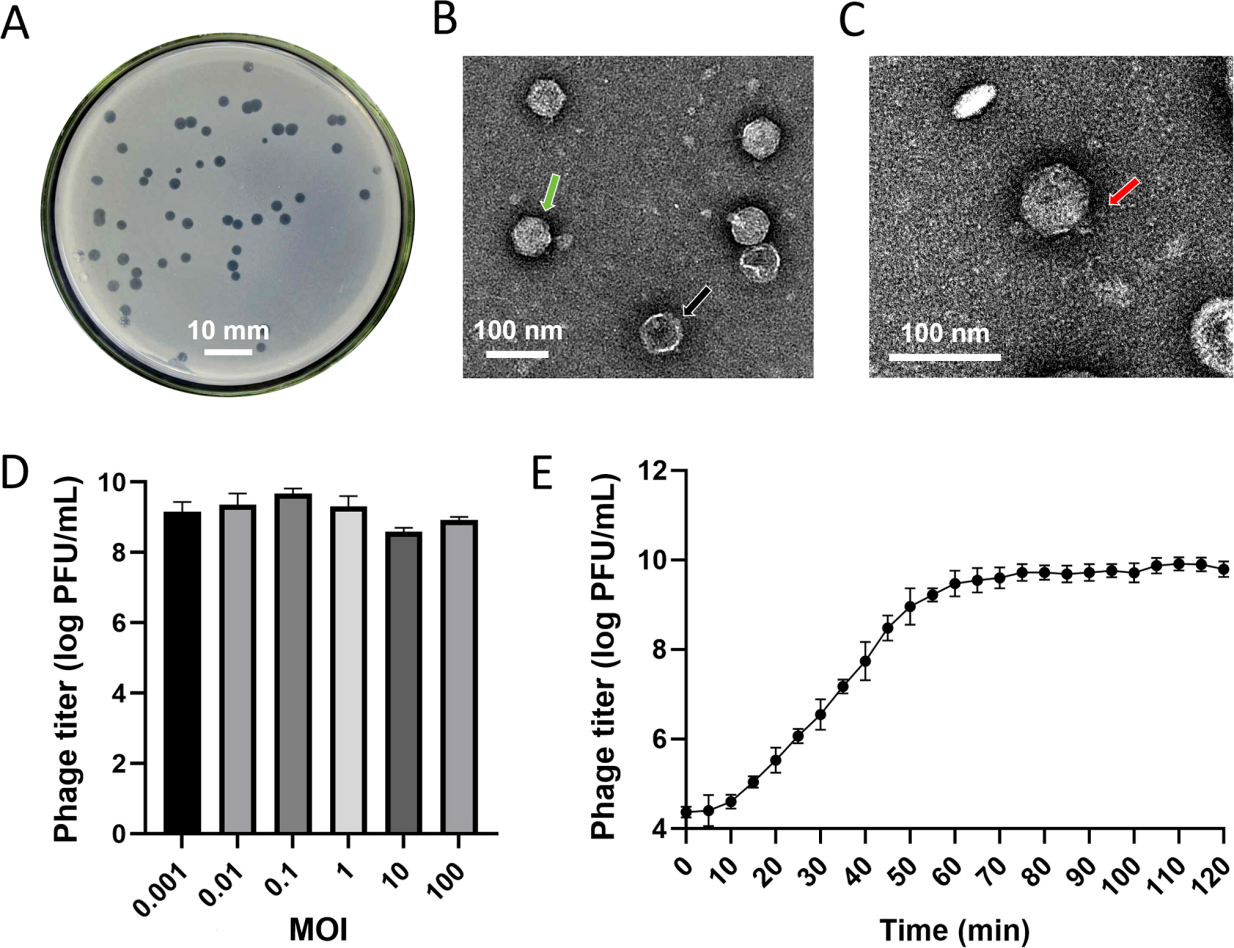
Biological characteristics of phage AbpL. **(A)** Plaque morphology of AbpL. Scale bar: 10 mm. **(B)** TEM morphology of AbpL. The green arrow indicates the complete virus particle, and the black arrow indicates the head with nucleic acid emptied. Scale bar: 100 nm. **(C)** Enlarged TEM morphology of AbpL, showing the short tail (indicated by the red arrow). Scale bar: 100 nm. **(D)** MOI determination of AbpL. **(E)** One-step growth curve of AbpL.

The plaques formed by the *A. baumannii* phage AbpL were predominantly transparent and approximately 2.0 mm in diameter, with a minority of smaller plaques measuring less than 1 mm (**Figure 1A**). Following lysis of the host bacteria, the phage titer in the lysate reached 1 × 10^10^ PFU/mL. Transmission electron microscopy revealed that phage AbpL possesses an icosahedral head with a diameter of approximately 63 nm (**Figure 1B**) and a short tail measuring about 9 nm in length (**Figure 1C**). The optimal multiplicity of infection (MOI) for phage AbpL was determined to be 0.1, as this condition yielded the highest phage titer (**Figure 1D**). One-step growth curve analysis indicated that phage AbpL has a short latent period of approximately 10 minutes, reaching a plateau approximately 60 minutes post infection (**Figure 1E**). The burst size, defined as the average number of phage particles released per infected cell, was estimated to be 169 for phage AbpL. Host range analysis demonstrated that phage AbpL could not lyse *E. coli*, *S. typhi*, *S. dysenteriae*, or *S. aureus*, but was capable of lysing 12 out of 23 (52%) clinical isolates of *A. baumannii* (**Supplementary Table S1**).

### 3.2 The stability of phage AbpL

**Figure 2:**
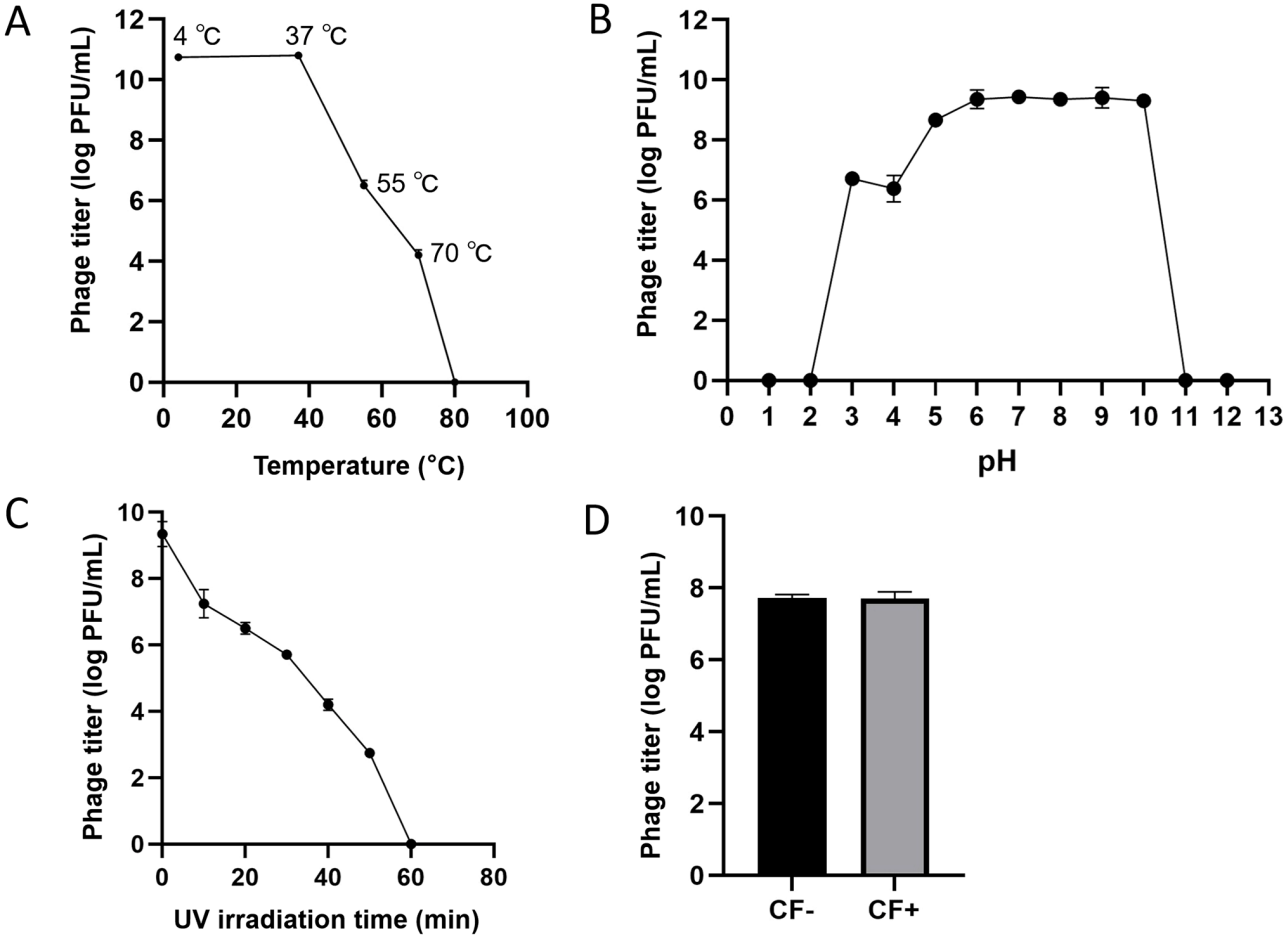
Physical and chemical stability of phage AbpL. **(A)** Temperature stability of AbpL. **(B)** pH stability of AbpL. **(C)** UV irradiation stability of AbpL. **(D)** Chloroform sensitivity of AbpL. CF: chloroform.

The phage AbpL remained stable at temperatures below 40°C for ten days. However, when the temperature increased from 40°C to 80°C, the phage titer gradually declined. Notably, AbpL retained a titer of 1 × 10^4^ PFU/mL even at 70°C. Upon exposure to temperatures above 80°C, the phage was completely inactivated (**Figure 2A**). AbpL exhibited high activity across a pH range of 3 to 10, with a titer exceeding 1 × 10^6^ PFU/mL under these conditions (**Figure 2B**). Prolonged UV exposure gradually reduced the phage titer, which eventually dropped to zero after 1 hour. Importantly, after approximately 30 minutes of ultraviolet (UV) irradiation, the titer remained above 1 × 10^5^ PFU/mL (**Figure 2C**), suggesting that standard UV disinfection may not effectively eliminate phage AbpL. Treatment with chloroform did not significantly alter the titer of AbpL (**Figure 2D**), indicating that the phage is insensitive to chloroform and that this reagent can be effectively used for phage AbpL purification.

### 3.3 Determination of AbpL genome termini

**Figure 3:**
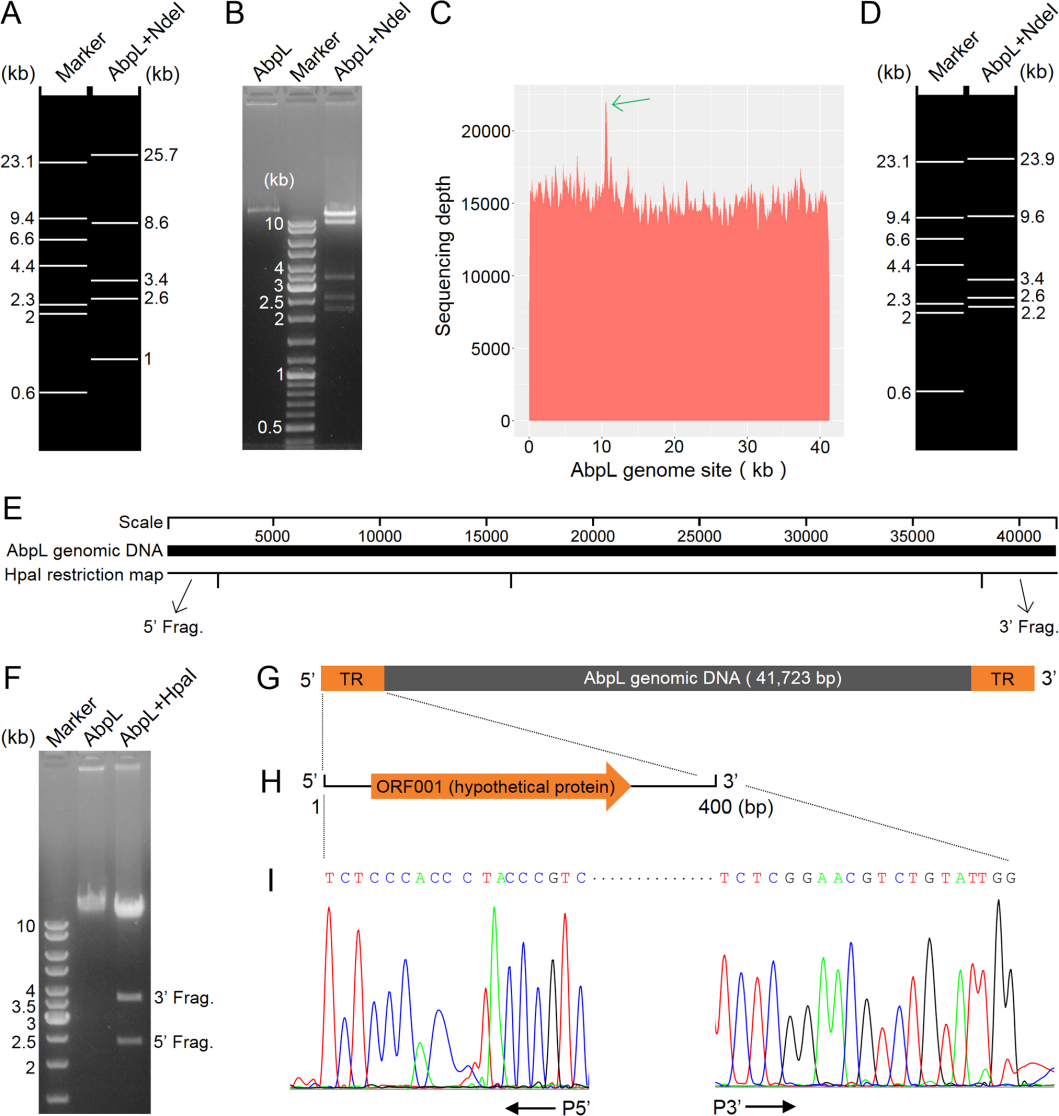
Genome termini analysis of AbpL. **(A)** Distribution of NdeI restriction fragments of the AbpL genome sequence after initial sequencing and assembly. **(B)** Electrophoretic pattern of NdeI-digested AbpL genomic DNA. **(C)** Sequencing depth plots across the AbpL genome. The green arrow indicates an abnormal sequencing depth peak. **(D)** Distribution of NdeI restriction fragments of the adjusted AbpL genome sequence. **(E)** Distribution of HpaI restriction sites in the adjusted AbpL genome sequence. The arrows indicate the 5’ end fragment (5’ Frag.) and the 3’ end fragment (3’ Frag.). **(F)** Electrophoretic profile of HpaI-digested AbpL genomic DNA. **(G)** Schematic representation of the intact AbpL genomic DNA, with orange regions at both termini indicating terminal repeats (TRs). **(H)** A protein with unknown function is encoded within the TR region. **(I)** Representative base peak chromatograms showing the terminal sequencing peaks and the termination sites.

The genomic DNA of phage AbpL was first sequenced and initially assembled as a circular molecule. The AbpL genome contains four NdeI restriction sites. When the genome was assumed to be linear, in silico digestion predicted five fragments (**Figure 3A**), which correspond to the bands observed in the actual NdeI digestion electrophoresis (**Figure 3B**). However, slight discrepancies were observed in the fragment sizes between the simulated and experimental digestion results. Analysis of the AbpL genome sequencing depth revealed an abnormal peak at approximately 11 kb (**Figure 3C**), suggesting the presence of terminal repeats. Based on this observation, the 11 kb sequence at the 5’ end of the genome was relocated to the 3’ end, and in silico digestion was performed again (**Figure 3D**), yielding results consistent with those of the experimental digestion (**Figure 3B**). Further analysis of the adjusted genome sequence revealed that the restriction enzyme HpaI could specifically cut both the 5’ and 3’ terminal fragments of the AbpL genome, which are of suitable size for separation by agarose gel electrophoresis (**Figure 3E**). HpaI digestion and subsequent electrophoresis confirmed that the 5’ and 3’ terminal fragments were approximately 2.4 kb and 3.6 kb in length, respectively (**Figure 3F**). Run-off sequencing of the terminal fragments enabled precise localization of the termini. Based on previously reported principles for determining phage genome termini (Lu et al., 2013), AbpL was confirmed to possess terminal redundancy (**Figure 3G**), with a duplicated region of 400 bp encoding a protein of unknown function (**Figure 3H**). The first nucleotide at the 5’ end of the terminal repeat is T, and the last nucleotide at the 3’ end is G (**Figure 3I**). Phylogenetic analysis revealed that the 400 bp terminal repeat sequence of phage AbpL exhibits homology across multiple *A. baumannii* phages (**Supplementary Figure S1**), indicating a potential conservation of the terminal genomic features among these phages.

### 3.4 The genome of phage AbpL

**Figure 4:**
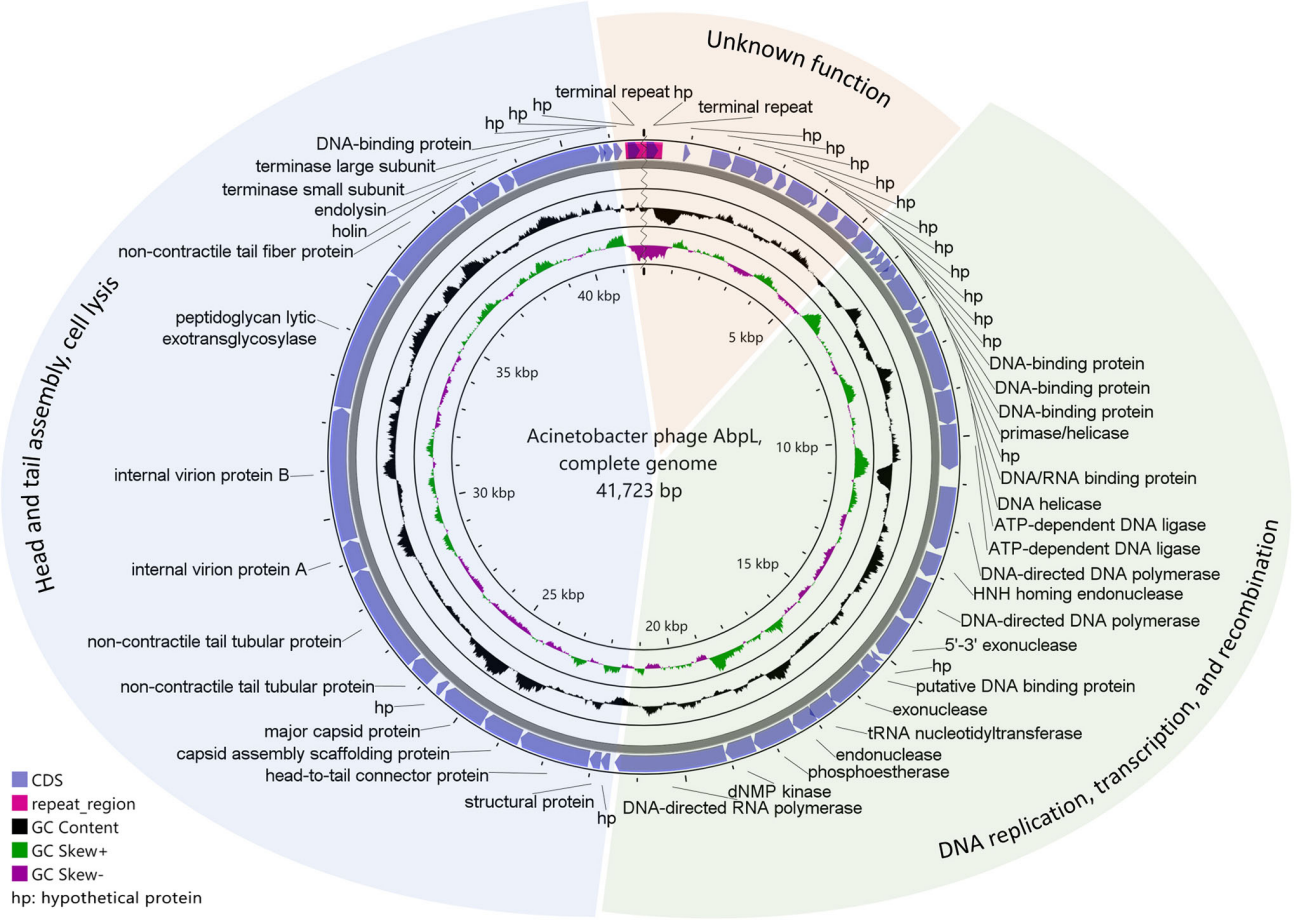
The genomic map of phage AbpL. The outermost circle represents genes encoded by the positive strand, followed by genes encoded by the negative strand. Different color blocks represent different functional modules within the AbpL genome.

The whole genome sequencing revealed that the total genome length of phage AbpL was 41,723 bp, with a G+C content of 39.35% (**Figure 4**). The AbpL genome encodes 53 proteins, among which 35 are predicted to perform specific functions, primarily including proteins involved in phage replication and regulation, structural proteins, and proteins associated with lysis and release, accounting for 66% of all encoded proteins (**Figure 4**). No tRNA-encoding genes were identified in the AbpL genome, nor were any genes related to drug resistance or virulence factors predicted. Based on the known functions of proteins and in accordance with the modular structural characteristics of the phage genomes (Lu et al., 2013), the AbpL genome was divided into three functional modules (**Figure 4**), among which the function of one module remained unclear. Additionally, DeePhage (Wu et al., 2021) was employed to predict the life cycle type of phage AbpL, and the analysis indicated that it is a lytic phage. The genome annotation data for AbpL (**Supplementary Table S3**) have been deposited in the NCBI GenBank database under the accession number OP171942.1.

### 3.5 The structural proteins of phage AbpL

**Figure 5:**
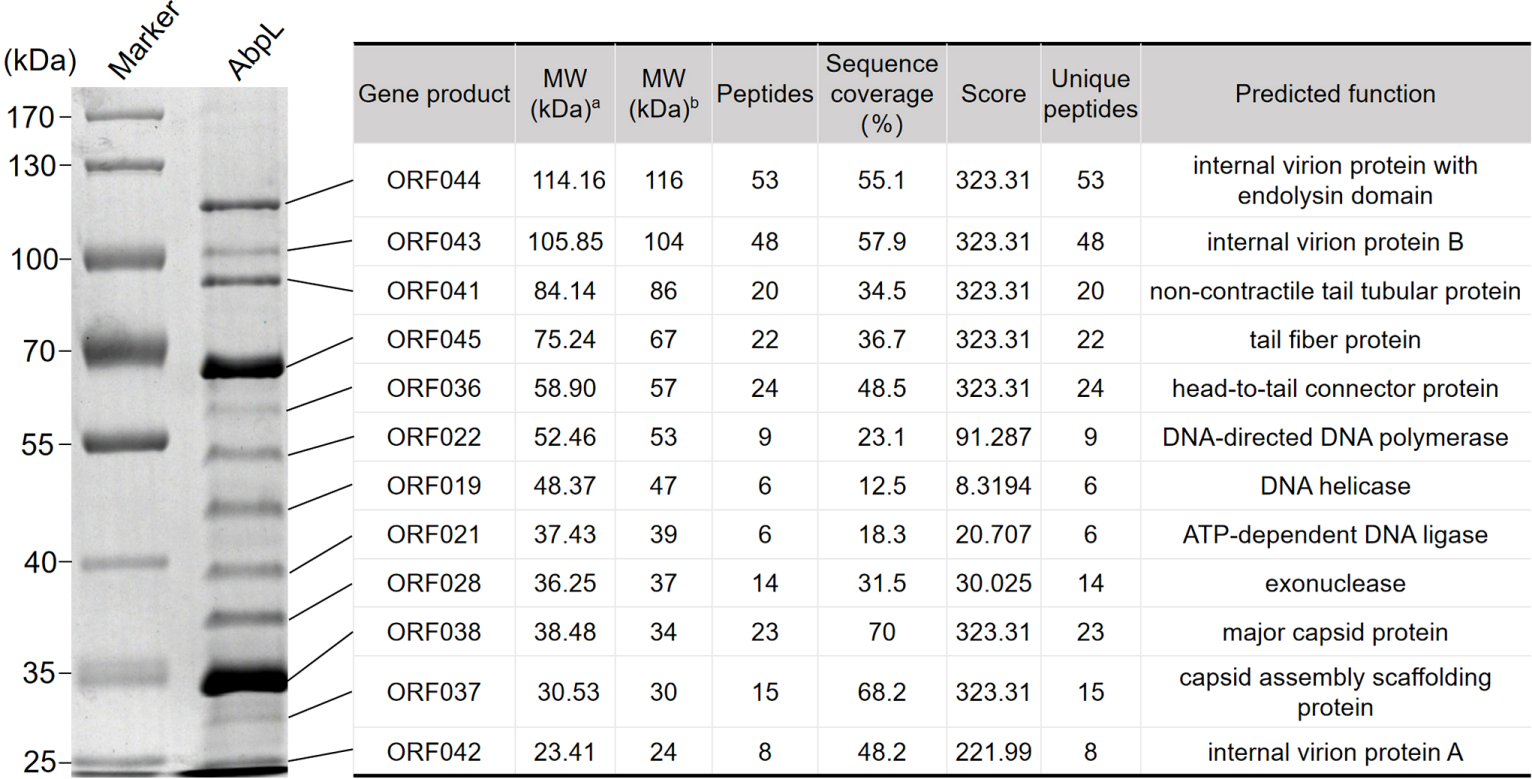
Identification of phage AbpL structural proteins. MW: molecular weight. ^a^MW value was theoretically calculated. ^b^MW value was experimentally estimated.

The structural proteins of phage AbpL were separated using SDS-PAGE, revealing approximately 13 distinct protein bands ranging from 25 kDa to 170 kDa (**Figure 5**). Following in-gel digestion, the proteins were analyzed by liquid chromatography-tandem mass spectrometry (LC-MS/MS), allowing the identification of 12 protein-coding genes, which were mapped to corresponding bands on the SDS-PAGE gel. The sequence coverage of these proteins, as determined by mass spectrometry analysis, exceeded 10% (**Figure 5**). The two most prominent protein bands corresponded to AbpL’s major capsid protein (ORF038, with a molecular weight of approximately 34 kDa) and the tail fiber protein (ORF045, with a molecular weight of approximately 67 kDa). Additionally, ORF019, ORF021, ORF022, and ORF028 were identified as DNA helicase, ATP-dependent DNA ligase, DNA-directed DNA polymerase, and exonuclease, respectively. These findings suggest that these proteins may function as internal viral components that are delivered into host cells along with the phage genome.

### 3.6 Comparative genomic analysis of phage AbpL

**Figure 6:**
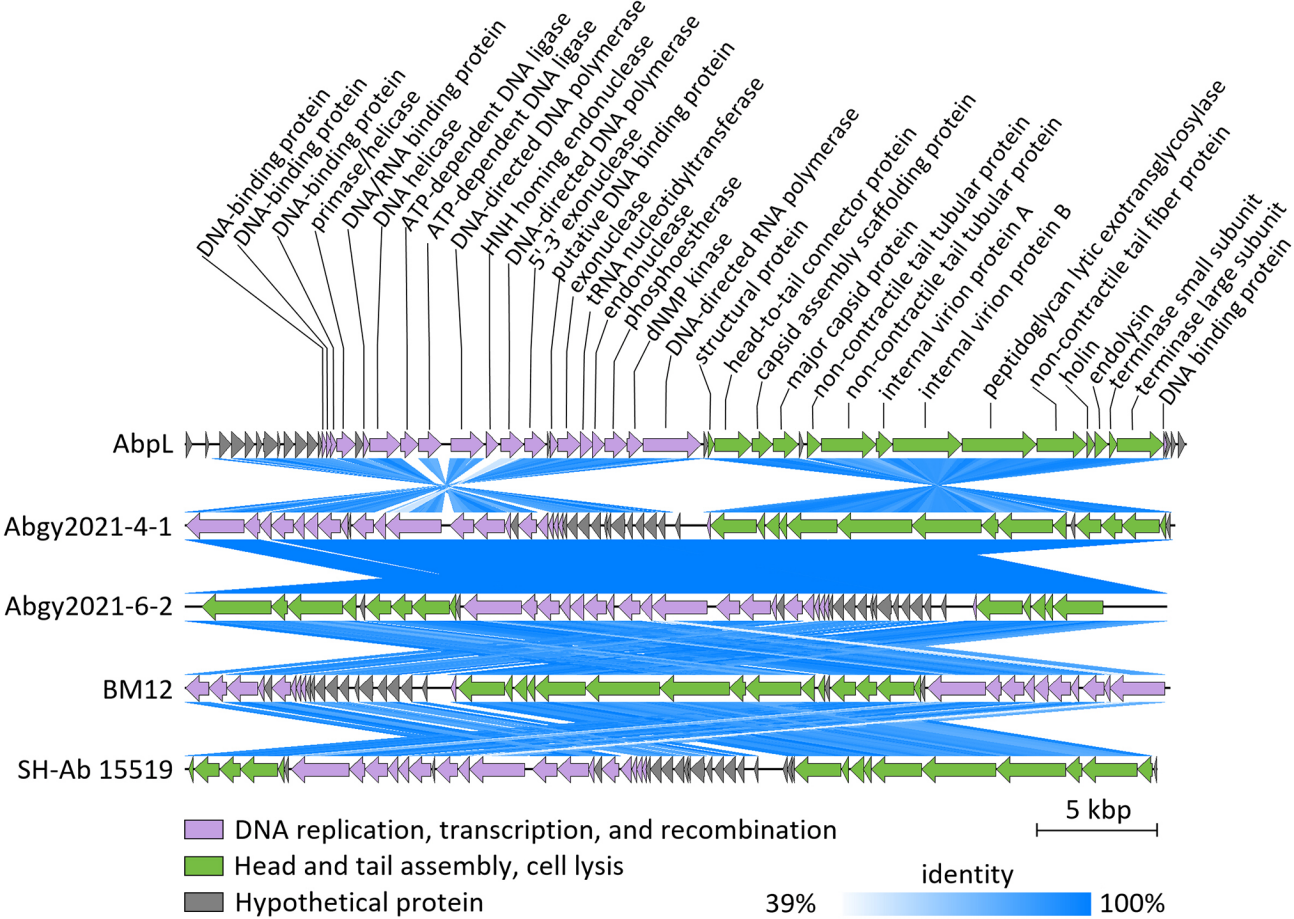
The genomic linear alignment analysis of phage AbpL. The similarity threshold for tBlastX comparison was set at 39%. The predicted gene functions were annotated above the corresponding open reading frames (ORFs) of phage AbpL.

The whole genome sequence of *A. baumannii* phage AbpL was analyzed using BLAST in NCBI (Singh and Raghava, 2016; Camacho et al., 2023), revealing that several phages exhibited sequence similarity to AbpL. Four phages with high similarity to AbpL were selected for tBlastX comparison against AbpL. The analysis revealed the presence of several conserved genomic regions among these 5 phages, although certain divergent regions were also observed (**Figure 6**). These divergent regions suggested the presence of insertions, deletions, translocations, inversions, and reverse complements across the phage genomes (**Figure 6**), which may represent mutations accumulated during long-term adaptation to their host bacteria. Notably, the genomic ends of these 5 phages were not identical, whereas the genomic ends of AbpL had been experimentally determined (**Figure 3**). Therefore, the genomic termini of the other phages required further validation. To better visualize the genomic sequence variations among these phages, a BRIG alignment was performed (**Supplementary Figure S2**). The results revealed that while these phages shared obvious similarities, they also exhibited distinct differences (represented as blank regions), which may reflect evolutionary recombination and selective pressures.

### 3.7 Phylogenetic and taxonomic analysis of phage AbpL

**Figure 7:**
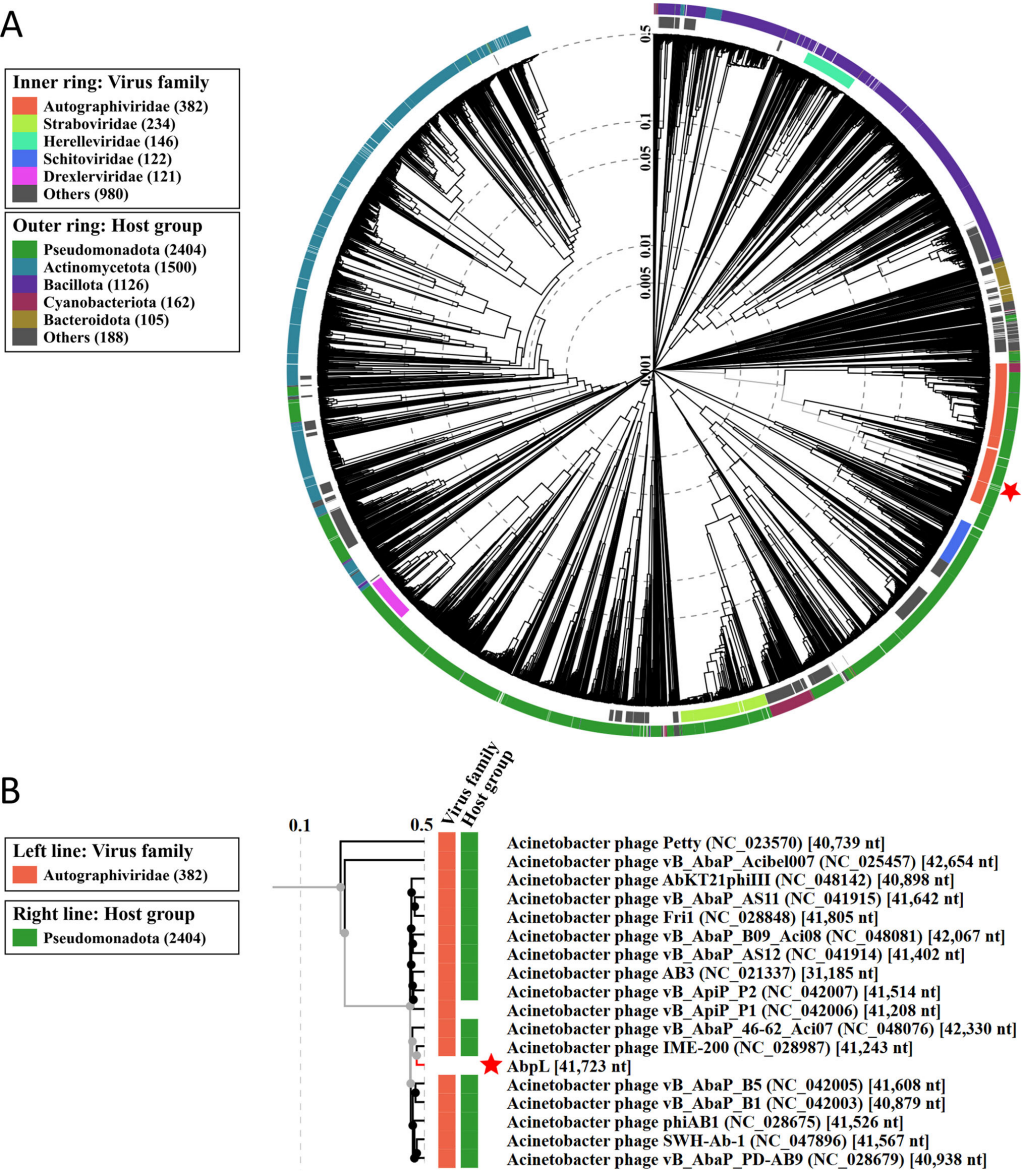
Proteomic phylogenetic tree of phage AbpL. **(A)** The circular viral proteomic tree of AbpL. **(B)** A portion of the rectangular proteomic tree illustrating the phages most closely related to AbpL.

Phylogenetic analysis based on the complete genome sequences revealed that phage AbpL was most closely related to *A. baumannii* phages pB3074 and vB_AbaP_ABWU2101 (**Supplementary Figure S3**). Phage AbpL showed a distinct genetic distance from other *Acinetobacter* phages, and genetically similar phages tended to cluster together, resulting in a phylogenetic tree with several branches. Within each branch, the G+C content of the phage genomes was relatively conserved (**Supplementary Figure S3**). Proteomic phylogenetic analysis of phage AbpL revealed that it clusters within the *Autographiviridae* family. This clade comprised 382 members, all of which located at a monophyletic group in the phylogenetic tree (**Figure 7A**). A closer examination of the proteomic phylogenetic tree indicated that phage AbpL is most closely related to *Acinetobacter* phage IME-200 (**Figure 7B**), a topology that differed from the evolutionary tree inferred from whole-genome sequences (**Supplementary Figure S3**). Taxonomic analysis using taxMyPhage revealed that phage AbpL shares average nucleotide identity (ANI) values exceeding 80% with 65 members of the *Friunavirus* genus (**Supplementary Table S4**), satisfying the International Committee on Taxonomy of Viruses (ICTV) criteria for genus-level classification. Based on the taxMyPhage analysis, phage AbpL should be classified as a member of the genus *Friunavirus*, subfamily *Beijerinckvirinae*, family *Autoscriptoviridae*, order *Autographivirales*, and class *Caudoviricetes*.

### 3.8 Phage AbpL exhibited strong *in vitro* bactericidal activity and effective biofilm removal capability

**Figure 8:**
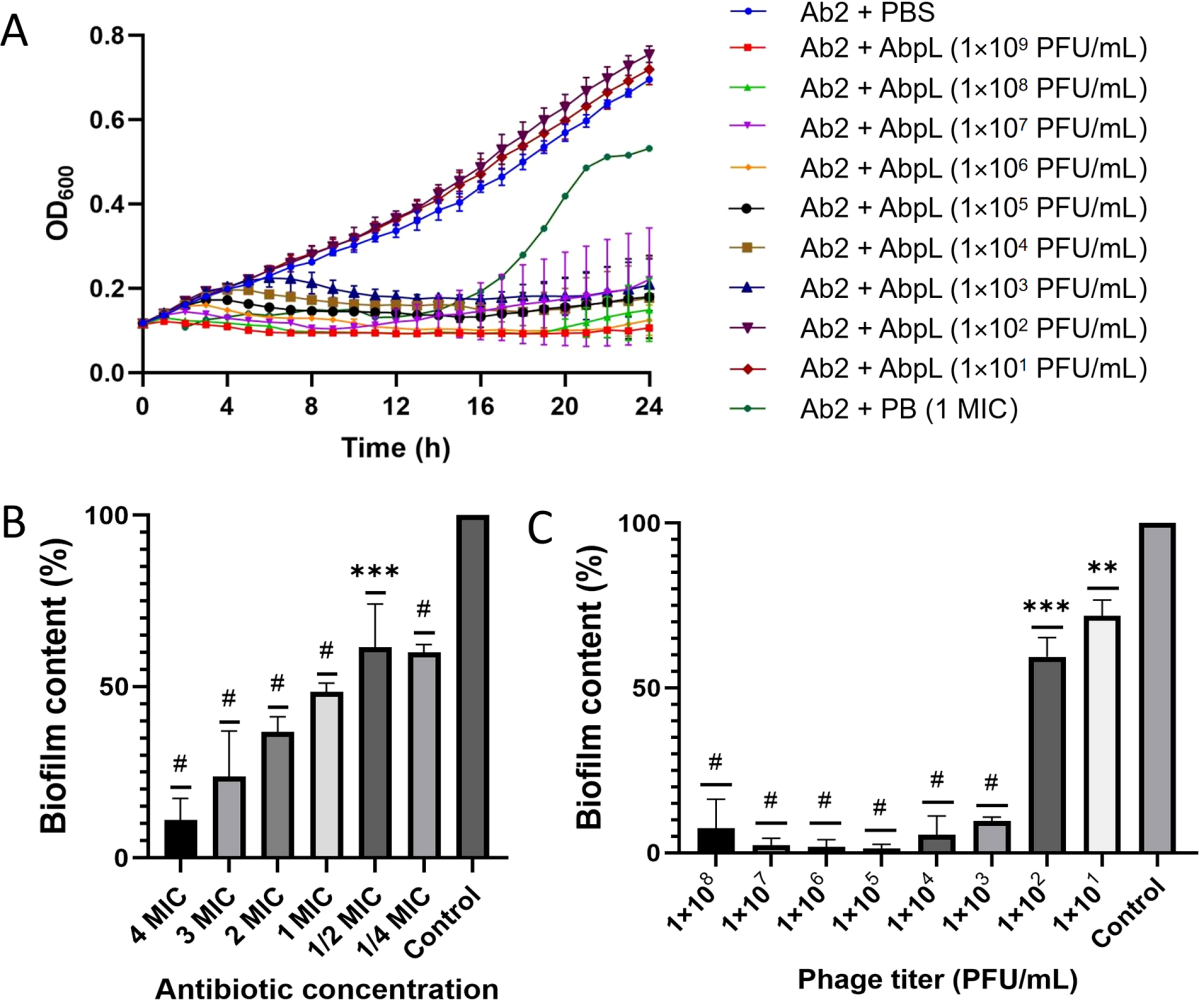
The time-kill curve of AbpL and biofilm removal test. **(A)** The time-kill curve of phage AbpL. PB: polymyxin B. 1 MIC: 8 μg/mL. **(B)** Biofilm removal by PB. 1 MIC: 8 μg/mL. **(C)** Biofilm removal by phage AbpL. ** indicates *P* < 0.01 compared with the control group; *** indicates *P* < 0.001 compared with the control group; # represents ****, indicating *P* < 0.0001 compared with the control group.

When the titer of phage AbpL reached 1 × 10^3^ PFU/mL or higher, it exhibited a significant bactericidal effect, and no regrowth of drug-resistant bacteria was observed within 24 hours (**Figure 8A**). The minimum inhibitory concentration (MIC) of polymyxin B (PB) against *A. baumannii* Ab2 was determined to be 8 μg/mL. The addition of PB at concentrations of 1 MIC effectively inhibited the early growth of Ab2. However, a significant increase in drug-resistant bacteria was observed after 14 hours of culture (**Figure 8A**). PB at concentrations of 1/4 MIC and above significantly inhibited biofilm formation by *A. baumannii* Ab2 (**Figure 8B**), and phage AbpL at titers of 1 × 10^1^ PFU/mL and above also showed significant biofilm inhibition (**Figure 8C**). When the titer of phage AbpL exceeded 1 × 10^3^ PFU/mL, more than 90% of Ab2 biofilm was eradicated, an effect comparable to that of PB at concentrations of 4 MIC.

### 3.9 Phage AbpL can effectively treat abdominal *A. baumannii* infection in mice

**Figure 9:**
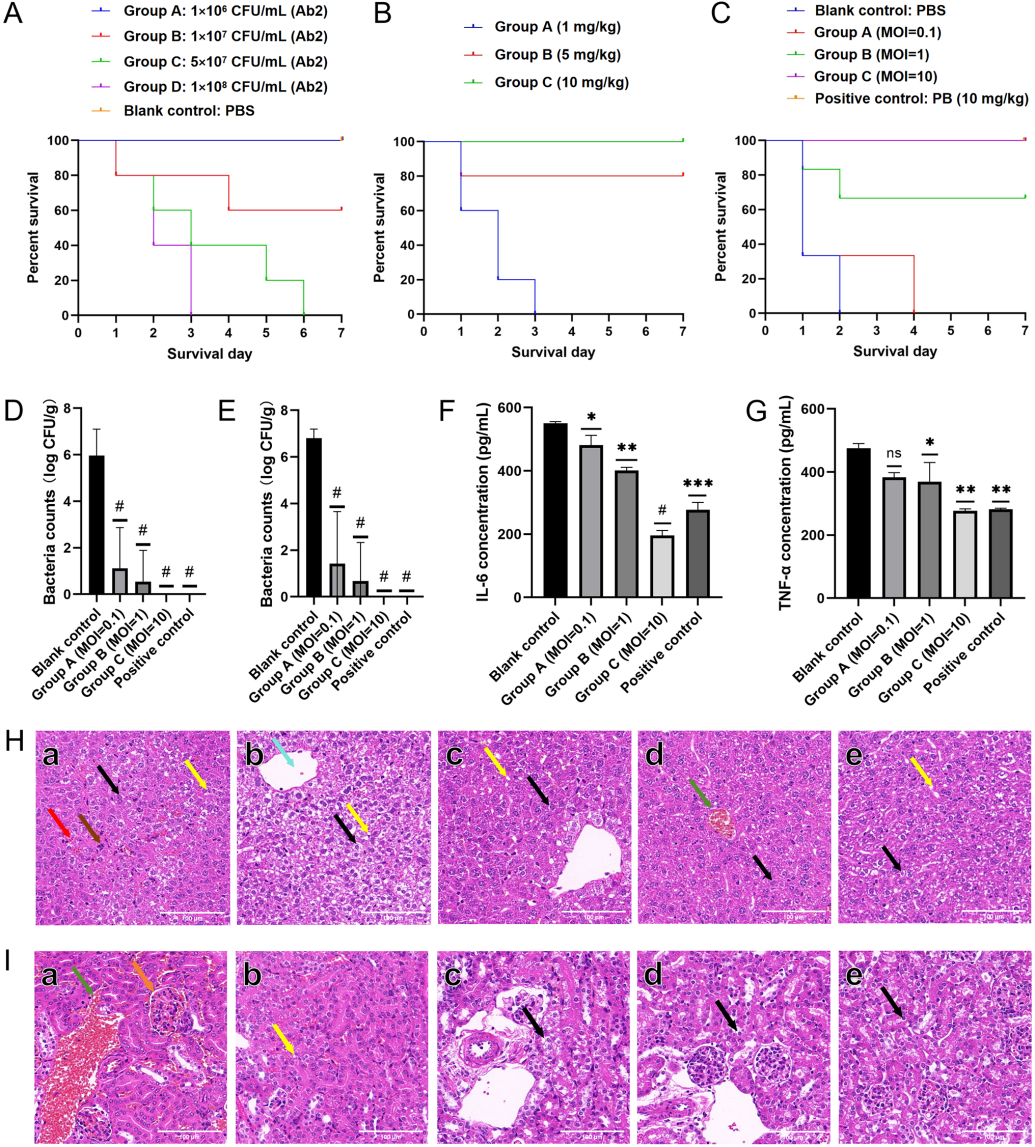
Study on the treatment of mouse abdominal cavity *A. baumannii* infection model with phage AbpL. **(A)** The survival curve of mice with abdominal cavity infections. **(B)** The effects of antibiotics at different concentrations in the treatment of mice with abdominal cavity infections. **(C)** The therapeutic effect of phage AbpL on mice with abdominal cavity infections. The injection doses of both bacteria and phage AbpL were 100 μL. The concentration of the bacterial suspension is approximately 1 × 10^8^ CFU/mL. The MOI values of 0.1, 1, and 10 correspond to the phage titers of 1 × 10^7^ PFU/mL, 1 × 10^8^ PFU/mL, 1 × 10^9^ PFU/mL, respectively. **(D)** Colony count in mouse liver after treatment. **(E)** Colony count in mouse kidneys after treatment. **(F)** The expression of IL-6 in the blood of mice after treatment. **(G)** The expression of TNF-α in the blood of mice after treatment. **(H)** Liver tissue sections before and after treatment in the mouse abdominal cavity infection model. a, blank control; b, the MOI of the injected phage AbpL is 0.1; c, the MOI of the injected phage AbpL is 1; d, the MOI of the injected phage AbpL was 10; e, positive control: PB. Black arrow: the cytoplasm of hepatocytes is loose and lightly stained; Red arrow: inflammatory cells; Brown arrow: hepatocyte necrosis; Yellow arrow: fat droplet; Green arrow: venous congestion; Blue arrow: vacuolated cytoplasm. **(I)** Renal tissue sections before and after treatment in the mouse model of abdominal cavity infections. a, blank control; b, the MOI of the injected phage AbpL is 0.1; c, the MOI of the injected phage AbpL is 1; d, the MOI of the injected phage AbpL was 10; e, positive control: PB. Green arrow: vascular congestion in the interstitium; Orange arrow: congestion of the capillary plexus within the glomerulus; Yellow arrow: tiny vacuoles in the cytoplasm; Black arrow: cytoplasmic loose light dye. The injection volume of PBS, Ab2, AbpL and PB was 100 μL per mouse. MOI: multiplicity of infection. Blank control: PBS; positive control: PB (10 mg/kg). ns: no significant difference; * indicates *P* < 0.05 compared with the blank control group; ** indicates *P* < 0.01 compared with the blank control group; *** indicates *P* < 0.001 compared with the blank control group; # represents ****, indicating *P* < 0.0001 compared with the blank control group.

A mouse septicemia model was established by intraperitoneal injection of *A. baumannii* Ab2. The survival of the mice was monitored and a survival curve was plotted (**Figure 9A**). Based on the observed survival rates, the minimum lethal dose of the host strain Ab2 was determined to be 1 × 10^8^ CFU/mL, which was subsequently used as the challenge dose in the experiments. Different doses of polymyxin B (PB) were tested for their therapeutic efficacy in the intraperitoneal infection model. Based on the survival data (**Figure 9B**), a dose of 10 mg/kg PB was selected for further use. Following treatment with phage AbpL at an MOI of 10, the survival rate of the infected mice reached 100% (**Figure 9C**), comparable to the effect observed in the PB-treated positive control group. To further evaluate the therapeutic efficacy of phage AbpL in treating Ab2-induced abdominal infection, bacterial loads in the liver and kidney were quantified. The results showed that different doses of phage AbpL significantly reduced the bacterial burden in both the liver (**Figure 9D**) and kidney (**Figure 9E**), indicating that AbpL effectively cleared *A. baumannii* Ab2 colonization in these organs. Analysis of inflammatory cytokines revealed that phage AbpL significantly reduced serum levels of interleukin-6 (IL-6) (**Figure 9F**) and tumor necrosis factor α (TNF-α) (**Figure 9G**) in infected mice, with a greater reduction observed compared to the PB treatment group.

Pathological examination revealed that a small number of hepatocytes in the blank control group exhibited hydropic degeneration, characterized by cellular swelling, cytoplasmic loosening, and pale staining. Mild hepatocyte steatosis was also observed, with the cytoplasm containing small, round vacuoles indicative of lipid droplets. Occasional hepatocyte necrosis and nuclear fragmentation were present, along with sparse inflammatory cell infiltration and disorganized hepatic cord architecture (**Figure 9H**). Following treatment with phage AbpL, hydropic degeneration and steatosis were markedly reduced in all experimental groups. No signs of hepatocyte necrosis were detected, and the inflammatory response was alleviated, indicating effective infection control (**Figure 9H**). The therapeutic effect was comparable to that observed in the PB treatment group. Histopathological analysis of kidney tissues showed that glomeruli in the renal cortex of the blank control group were uniformly distributed, although glomerular capillary plexus congestion and interstitial vascular congestion were frequently observed (**Figure 9I**). In all treatment groups, renal tissue damage was ameliorated, and no telangiectasia was detected (**Figure 9I**).

## 4 Discussions

*A. baumannii* is particularly notorious for its exceptional capacity to acquire antimicrobial resistance (Tan et al., 2023; Ghahramani et al., 2024). Therefore, there is an urgent need to develop safer and more effective strategies to combat drug-resistant strains of this pathogen (Martins-Sorenson et al., 2020). Phages, as natural agents capable of targeting and lysing bacteria, have gradually gained attention. These viruses specifically destroy bacterial cells without significantly affecting mammalian cells (Elshamy et al., 2025), and various clinical trials as well as developments in novel therapies are actively underway. However, due to the limited number of phages currently isolated and characterized, there is an urgent need to identify and isolate numerous new phages with high lytic efficiency. This effort would contribute to the establishment of a comprehensive phage library, serving as a valuable resource for future antibacterial interventions. Although nearly 500 A*. baumannii* phages have been deposited in the GenBank database, this number is still insufficient for constructing a fully representative phage library. In cases of acute infection, the time required to develop a personalized treatment plan is often too long. Therefore, isolating and characterizing a large number of phages in advance is essential to provide a solid foundation for phage therapy.

*A. baumannii* phages exhibit potent bactericidal activity, a key attribute for effective antimicrobial agents. For example, phage Abgy202141 achieves over 90% adsorption within 5 minutes post-infection and undergoes rapid replication, reaching peak progeny yield by approximately 45 minutes (Tian et al., 2024). Phage Pϕ-Bw-Ab has a latent period of ~50 minutes, and lysis occurs between 50 and 60 minutes post-infection (Rahimzadeh Torabi et al., 2021). In contrast, phage pIsf-AB02 displays a shorter latent period of ~30 minutes, followed by a lysis phase lasting ~70 minutes (Sisakhtpour et al., 2022). Notably, the phage AbpL isolated in this study exhibited a short latent period of ~10 minutes and a lysis period of ~50 minutes, placing it among the fastest-lysing *A. baumannii* phages reported to date—comparable to Abgy202141 in kinetic profile. Phage stability under varying environmental conditions is another critical parameter for therapeutic applicability. Many *A. baumannii* phages retain high infectivity across broad temperature and pH ranges. For instance, phage Ab_WF01 shows optimal activity at 25°C and remains stable up to 50°C, with its titer declining to ~35% at 70°C; it also maintains robust activity within a pH range of 5–10 (Wang et al., 2024b). Similarly, phage HZY2308 demonstrates maximal activity between 4°C and 50°C and across pH 5–9 (Wang et al., 2024a). In this study, phage AbpL remained stable below 37°C, with a linear decrease in stability above this threshold. At 70°C, infectious titer dropped to ~38%, and complete inactivation was observed at −80°C. AbpL retained full infectivity within pH 6–10, a stability profile comparable to that of Ab_WF01 and HZY2308. Collectively, the rapid infection kinetics and favorable environmental stability of phage AbpL underscore its strong potential as a candidate for combating drug-resistant *A. baumannii* infections.

So far, although numerous phages have been isolated and characterized globally, many aspects regarding phage genes and life cycles remain poorly understood. This lack of knowledge limits the direct and widespread application of natural phages in clinical settings. Therefore, the analysis of phage genomes provides a crucial foundation for disease treatment, environmental regulation, and synthetic biology. In this study, the complete genome of phage AbpL was sequenced and analyzed, revealing a 400-bp direct repeats at its genomic DNA termini. Terminal direct repeats represent a form of terminal redundancy commonly found in phage genomes and may play a role in genome cyclization or replication through recombination mechanisms (Beaulaurier et al., 2020). The length of terminal direct repeats varies among different phages. For instance, the *Pseudomonas aeruginosa* phage PaP1 contains an 1190-bp terminal direct repeat (Lu et al., 2013), whereas the Listeria phage A511 genome features a 3125-bp direct repeat (Klumpp et al., 2008). The terminal regions of most phage genomes remain inaccurately characterized, leading to substantial variability in the annotated terminal positions of genomes from closely related phages within the same genus in public databases. Phages that are evolutionarily closely related, particularly those within the same genus, typically exhibit conserved terminal redundancy patterns. This study accurately determined the terminal redundancy sequence of phage AbpL, providing a valuable reference for genome annotation of closely related phages within the *Friunavirus* genus. Furthermore, elucidating the structure of phage genomic termini is of significant importance in synthetic biology, as it enables the artificial synthesis and reactivation of phages and supports the development of chassis phages for engineered applications (Yuan et al., 2022).

The genome of phage AbpL encodes a total of 53 proteins, 18 of which have currently unknown functions and will serve as a focus for future research. Notably, phage AbpL encodes endolysin-related proteins, making it a promising candidate for further investigation. Compared to conventional phages and antibiotics, endolysins and depolymerases encoded by phages exhibit higher specificity and a lower likelihood of inducing drug resistance (Abdelaziz et al., 2021), thereby offering a novel and promising alternative for phage therapy. Currently, numerous studies have explored the use of bacteriolytic enzymes in treating multidrug-resistant bacterial infections. For example, recombinant endolysin LysSS has demonstrated activity against multidrug-resistant *A. baumannii*, *E. coli*, *Klebsiella pneumoniae*, *P. aeruginosa*, and *Salmonella* (Kim et al., 2020). LysJEP8, a novel endolysin derived from the *E. coli* phage JEP8, exhibits significant antibacterial activity against key Gram-negative members of the ESKAPE pathogens (*Enterococcus faecium*, *S. aureus*, *K. pneumoniae*, *A. baumannii*, *P. aeruginosa*, and *Enterobacter* species) (Carratalá et al., 2024). Determining the complete genome sequence of a phage enables phage modification and the synthesis of engineered phages (Baker et al., 2025), which can expand host range and enhance lytic efficiency. In the ongoing “arms race” between phages and bacteria, bacteria have evolved various defense mechanisms to interfere with phage genome replication (Rodriguez-Rodriguez et al., 2025). By incorporating functional genes into the phage genome, the efficiency of phage infection can be improved. Additionally, the removal of virulence genes and non-essential functional elements allows the utilization of a phage chassis genome to achieve a highly controllable and biosafe therapeutic platform (Sun et al., 2023).

Phage therapy can be categorized into single-strain phage therapy, phage cocktail therapy, combination therapy involving phages and other antimicrobial agents, and phage derivative therapy. Research indicated that due to the continuous evolution and mutation of bacteria, the use of single phage therapy carries a high risk of developing phage resistance (Strathdee et al., 2023). Therefore, a limitation of this study is its exclusive focus on evaluating the efficacy of single-strain therapy using the natural phage AbpL, without addressing phage cocktail strategies, combination therapies, or engineered phage applications. These areas represent promising directions for future research. *A. baumannii* can cause a range of infections, including septicemia, urinary tract infections, meningitis, and pneumonia (Whiteway et al., 2021; Li et al., 2025). It is essential to evaluate the effectiveness of phage therapy in animal models during preclinical research before advancing it as a viable and reliable treatment option for *A. baumannii* infections in humans. Previous studies have conducted extensive animal experiments on *A. baumannii* infection, such as assessing survival rates, bacterial loads, and inflammatory markers in bumblebee larval and murine infection models (Li et al., 2023), which have confirmed the *in vivo* antibacterial efficacy of certain *A. baumannii* phages. This study further underscores the therapeutic potential of phage therapy against drug-resistant *A. baumannii* infections by demonstrating the efficacy of phage AbpL in a murine model of sepsis induced by intraperitoneal infection, thereby making a meaningful contribution to the advancement of *A. baumannii* phage therapy. Although phages hold great promise for treating bacterial infections, substantial progress is still needed in various aspects of phage research, including the isolation and identification of novel phages, resource development, formulation of phage cocktails, development of personalized therapies, and advances in phage synthetic biology. Investigating the interactions between phages and bacteria also holds long-term significance for achieving efficient phage therapy against drug-resistant bacterial infections in the future.

## 5 Conclusions

In this study, the lytic phage AbpL was isolated from sewage in Chongqing, China. It is capable of specifically lysing 52% of clinical *A. baumannii* isolates, including the host strain Ab2. This short-tailed phage possesses an icosahedral head, a linear double-stranded DNA genome of 41,723 base pairs, and a 400-bp direct terminal repeat. It does not encode any antibiotic resistance or virulence genes. Phage AbpL exhibited a short latent period, strong lytic activity, and high tolerance to variations in temperature, pH, and UV conditions. AbpL also demonstrated effective biofilm removal capability. In a murine septicemia model, intraperitoneal administration of AbpL at an MOI of 10 significantly improved mouse survival rates and reduced bacterial loads as well as inflammatory cytokine levels in the liver and kidneys, thereby confirming its potent antibacterial efficacy both *in vitro* and *in vivo*.

## Data Availability

The datasets presented in this study can be found in online repositories. The names of the repository/repositories and accession number(s) can be found in the article/**Supplementary Material**.
