# Supplementary material for: Characterization of phage AbpL with a terminally redundant genome and its therapeutic potential against drug-resistant Acinetobacter baumannii infections
Source: Front Cell Infect Microbiol. 2026 Feb 3;16:1760018. doi: 10.3389/fcimb.2026.1760018 (PMC12960631; doi:10.3389/fcimb.2026.1760018)
Supplement: Supplementary file 2 [file DataSheet2.pdf]

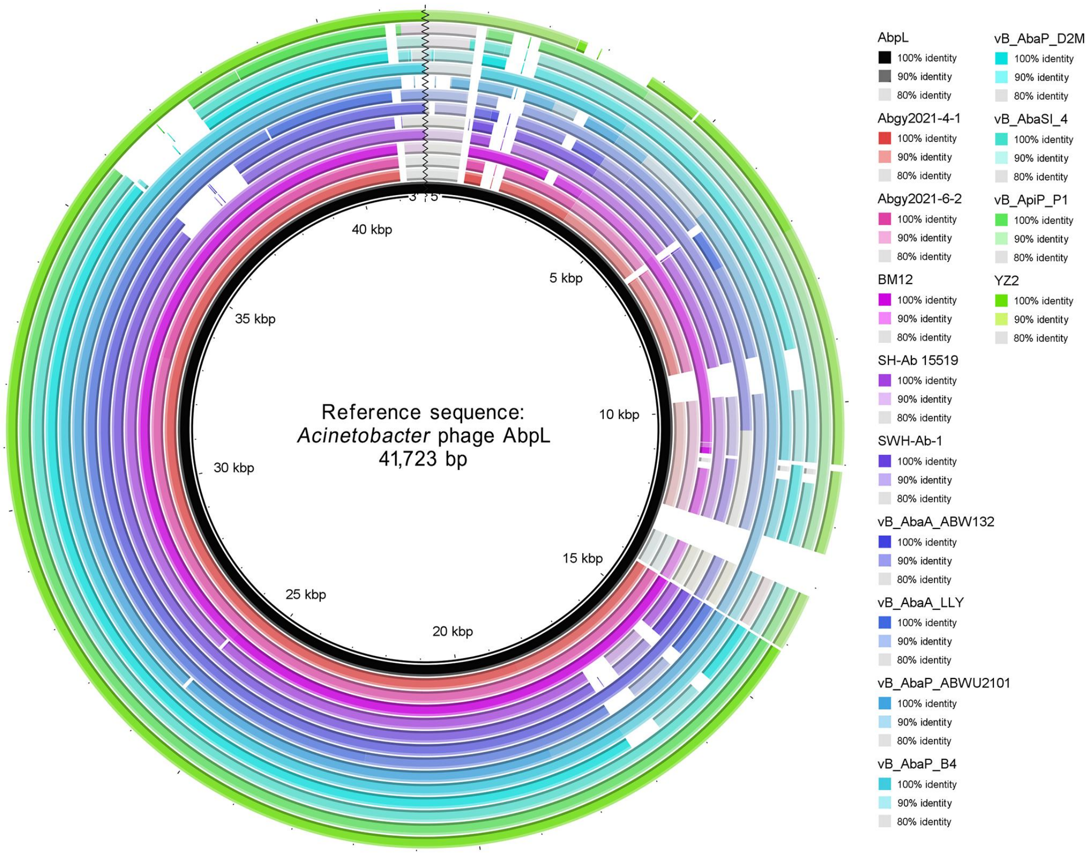

Figure S2. BRIG comparison of complete genome sequences of AbpL with other 13 related phages showed in Figure 6. Identity labels of the 14 phage genomes are shown in the same order as the rings from the innermost to the outermost.
