# Supplementary material for: Characterization of phage AbpL with a terminally redundant genome and its therapeutic potential against drug-resistant Acinetobacter baumannii infections
Source: Front Cell Infect Microbiol. 2026 Feb 3;16:1760018. doi: 10.3389/fcimb.2026.1760018 (PMC12960631; doi:10.3389/fcimb.2026.1760018)
Supplement: Supplementary file 3 [file DataSheet3.pdf]

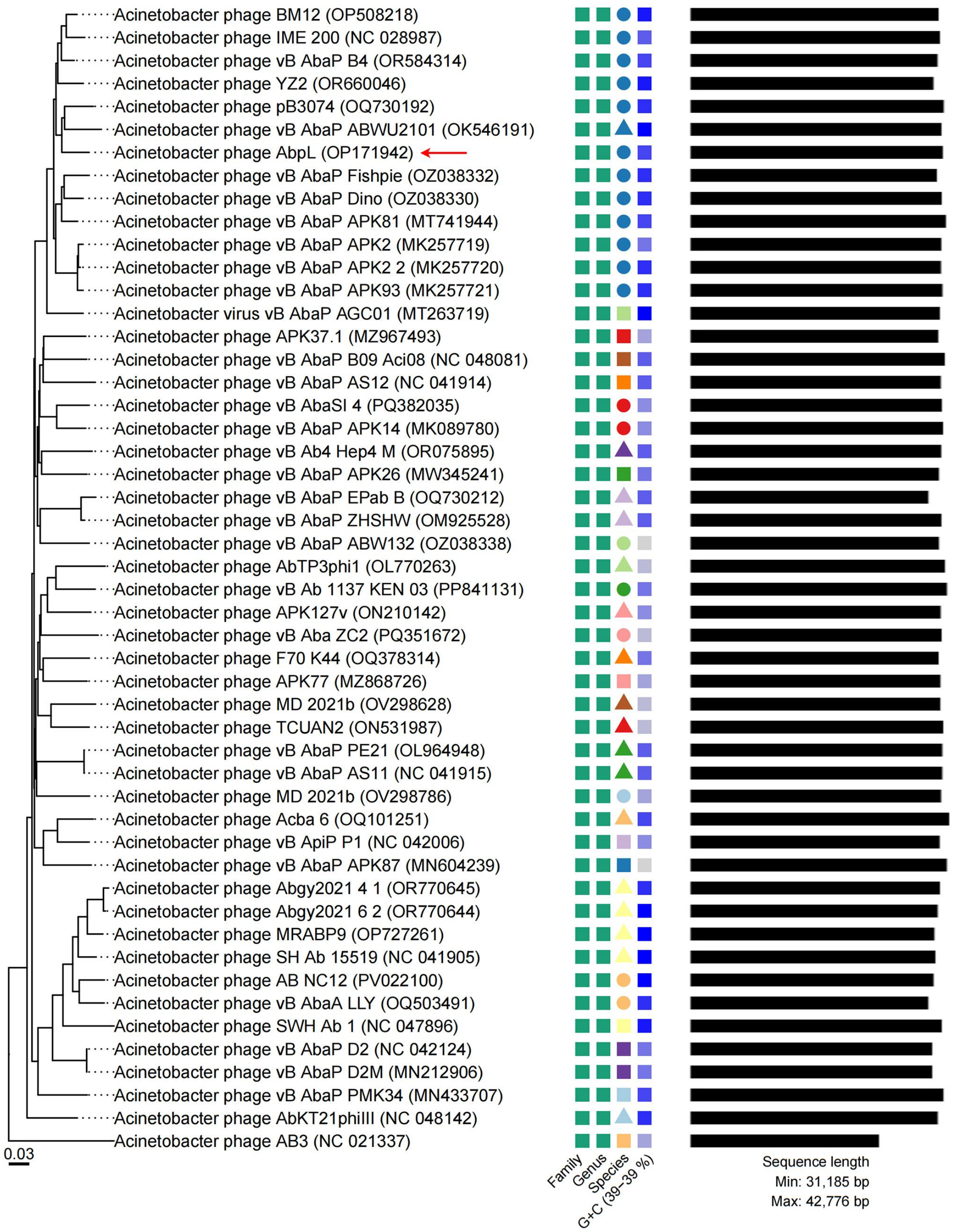

Figure S3. The phylogenetic tree of phage AbpL based on whole genome sequences. The scale bar indicates the relative genetic distance. All phages shown belong to the same family and genus, and are therefore represented by green squares in the figure. Species: different shapes and colors indicate the degree of similarity or divergence among phages. G+C: the shade intensity of the squares reflects variations in genomic

G+C content. Black rectangles: represent the relative lengths of phage genomes.
