# Supplementary material for: Characterization of phage AbpL with a terminally redundant genome and its therapeutic potential against drug-resistant Acinetobacter baumannii infections
Source: Front Cell Infect Microbiol. 2026 Feb 3;16:1760018. doi: 10.3389/fcimb.2026.1760018 (PMC12960631; doi:10.3389/fcimb.2026.1760018)
Supplement: Supplementary file 4 [file Table1.docx]

Table S1. Lysis of different bacterial strains by AbpL

| Number | Strains | Source* | Multidrug resistance | AbpL |
| --- | --- | --- | --- | --- |
| 1 | *Acinetobacter baumannii* Ab2 | Burn Department of Southwest Hospital, Army Medical University  (Clinical isolate) | Y | + |
| 2 | *Acinetobacter baumannii* Ab16716 | Laboratory Department, The Second Affiliated Hospital of Chongqing Medical  University  (Clinical isolates) | Y | - |
| 3 | *Acinetobacter baumannii* Ab16743 |  | Y | + |
| 4 | *Acinetobacter baumannii* Ab16812 |  | Y | - |
| 5 | *Acinetobacter baumannii* Ab16819 |  | Y | - |
| 6 | *Acinetobacter baumannii* Ab16835 |  | Y | + |
| 7 | *Acinetobacter baumannii* Ab16868 |  | Y | + |
| 8 | *Acinetobacter baumannii* Ab16886 |  | Y | + |
| 9 | *Acinetobacter baumannii* Ab16891 |  | Y | - |
| 10 | *Acinetobacter baumannii* Ab16900 |  | Y | - |
| 11 | *Acinetobacter baumannii* Ab17004 |  | Y | + |
| 12 | *Acinetobacter baumannii* Ab17006 |  | Y | + |
| 13 | *Acinetobacter baumannii* Ab17008 |  | Y | - |
| 14 | *Acinetobacter baumannii* Ab17023 |  | Y | - |
| 15 | *Acinetobacter baumannii* Ab17035 |  | Y | + |
| 16 | *Acinetobacter baumannii* Ab17111 |  | Y | - |
| 17 | *Acinetobacter baumannii* Ab17133 |  | Y | + |
| 18 | *Acinetobacter baumannii* Ab17161 |  | Y | - |
| 19 | *Acinetobacter baumannii* Ab17162 |  | Y | - |
| 20 | *Acinetobacter baumannii* Ab17163 |  | Y | + |
| 21 | *Acinetobacter baumannii* Ab17225 |  | Y | - |
| 22 | *Acinetobacter baumannii Ab17240* |  | Y | + |
| 23 | *Acinetobacter baumannii* Ab17291 |  | Y | + |
| 24 | *Escherichia coli* O157:H7 | Department of Microbiology, College of Basic Medical Sciences, Army Medical University  (Laboratory preservation) | N | - |
| 25 | *Shigella dysenteriae*  G1.126 |  | N | - |
| 26 | *Salmonella enterica serovar Typhi* Ty2 |  | N | - |
| 27 | *Staphylococcus aureus* Newman |  | N | - |

*All located in Chongqing, China; Y: yes; N: no; +: lysis; -: no lysis.
