# Supplementary material for: Characterization of phage AbpL with a terminally redundant genome and its therapeutic potential against drug-resistant Acinetobacter baumannii infections
Source: Front Cell Infect Microbiol. 2026 Feb 3;16:1760018. doi: 10.3389/fcimb.2026.1760018 (PMC12960631; doi:10.3389/fcimb.2026.1760018)
Supplement: Supplementary file 6 [file Table3.docx]

| Table S3. Genome annotations of phage AbpL | | | | |
| --- | --- | --- | --- | --- |
| **Query name** | **Location (bp; from..to)** | **Query length (aa)** | **Identity** | **Best hit description** |
| ORF001 | 57..314 | 85 | 80.95% | hypothetical protein  (*Acinetobacter* virus vB_AbaP_AGC01) |
| ORF002 | 869..1012 | 47 | * | hypothetical protein |
| ORF003 | 1461..1931 | 156 | 99.36% | hypothetical protein  (*Acinetobacter* phage vB_AbaP_APK81) |
| ORF004 | 1960..2511 | 183 | 99.45% | hypothetical protein  (*Acinetobacter* phage vB_AbaP_ZHSHW) |
| ORF005 | 2513..2887 | 124 | 98.39% | hypothetical protein  (*Acinetobacter* phage APK16) |
| ORF006 | 2979..3212 | 77 | 98.70% | hypothetical protein  (*Acinetobacter* phage vB_AbaP_PD-AB9) |
| ORF007 | 3287..3883 | 198 | 99.49% | hypothetical protein  (*Acinetobacter* phage AbTP3phi1) |
| ORF008 | 3871..3984 | 37 | 94.59% | hypothetical protein  (*Acinetobacter* phage APK20) |
| ORF009 | 4143..4541 | 132 | 98.48% | hypothetical protein  (*Acinetobacter* phage Paty) |
| ORF010 | 4620..5108 | 162 | 99.38% | hypothetical protein  (*Acinetobacter* phage Fri1) |
| ORF011 | 5110..5547 | 145 | 98.62% | hypothetical protein  (*Acinetobacter* phage IME-200) |
| ORF012 | 5558..5725 | 55 | 98.18% | hypothetical protein  (*Acinetobacter* phage vB_ApiP_P1) |
| ORF013 | 5712..5900 | 62 | 71.43% | DNA-binding protein  (*Acinetobacter* virus vB_AbaP_AGC01) |
| ORF014 | 5897..6115 | 72 | 46.75% | DNA-binding protein  (*Acinetobacter* phage AB_SZ6) |
| ORF015 | 6057..6329 | 90 | 81.11% | DNA-binding protein  (*Acinetobacter* phage YZ2) |
| ORF016 | 6319..7119 | 266 | 99.62% | primase/helicase  (*Acinetobacter* phage vB_AbaP_APK2) |
| ORF017 | 7119..7436 | 105 | 100% | hypothetical protein  (*Acinetobacter* phage vB_AbaP_W8) |
| ORF018 | 7436..7678 | 80 | 83.54% | DNA/RNA binding protein  (*Acinetobacter* phage vB_AbaP_AS11) |
| ORF019 | 7691..8989 | 432 | 99.54% | DNA helicase  (phage vB_AbaP_B09_Aci08) |
| ORF020 | 8992..9729 | 245 | 95.92% | ATP-dependent DNA ligase  (*Acinetobacter* phage YZ2) |
| ORF021 | 9726..10709 | 327 | 94.80% | ATP-dependent DNA ligase  (*Acinetobacter* phage vB_AbaP_ABWU2101) |
| ORF022 | 11077..12456 | 459 | 97.60% | DNA-directed DNA polymerase  (*Acinetobacter* phage AbKT21phiIII) |
| ORF023 | 12553..13080 | 175 | 100% | HNH homing endonuclease  (*Acinetobacter* phage APK20) |
| ORF024 | 13166..14104 | 312 | 99.68% | DNA-directed DNA polymerase  (*Acinetobacter* phage AB_SZL2) |
| ORF025 | 14148..15038 | 296 | 98.65% | 5'-3' exonuclease  (*Acinetobacter* phage SWH-Ab-1) |
| ORF026 | 15095..15214 | 39 | 100.00% | hypothetical protein  (*Acinetobacter* phage Abp1) |
| ORF027 | 15205..15543 | 112 | 90.91% | putative DNA binding protein  (*Acinetobacter* phage vB_AbaP_AS12) |
| ORF028 | 15524..16489 | 321 | 98.44% | exonuclease  (*Acinetobacter* phage vB_AbaP_ZHSHW) |
| ORF029 | 16470..17048 | 192 | 96.83% | tRNA nucleotidyltransferase (*Acinetobacter* phage vB_AbaP_46-62_Aci07) |
| ORF030 | 16979..17485 | 168 | 99.35% | Endonuclease  (*[Acinetobacter](https://blast.ncbi.nlm.nih.gov/Blast.cgi" \l "alnHdr_WUV29641" \o "Go to alignment for endonuclease VII [Acinetobacter phage vB_AbaP_W8] >emb\|CAL1777016.1\| endonuclease VII [Acinetobacter phage vB_AbaP_ABW132])* [phage vB_AbaP_W8](https://blast.ncbi.nlm.nih.gov/Blast.cgi" \l "alnHdr_WUV29641" \o "Go to alignment for endonuclease VII [Acinetobacter phage vB_AbaP_W8] >emb\|CAL1777016.1\| endonuclease VII [Acinetobacter phage vB_AbaP_ABW132])) |
| ORF031 | 17489..18424 | 311 | 99.68% | phosphoestherase with HTH domain (*Acinetobacter* phage vB_AbaP_B09_Aci08) |
| ORF032 | 18424..19074 | 216 | 99.54% | dNMP kinase  (*Acinetobacter* phage vB_AbaP_APK2-2) |
| ORF033 | 19083..21500 | 805 | 99.88% | DNA-directed RNA polymerase  (*Acinetobacter* phage BM12) |
| ORF034 | 21610..21807 | 65 | 96.92% | hypothetical protein (*Salmonella enterica*) |
| ORF035 | 21804..22055 | 83 | 98.80% | structural protein  (*Acinetobacter* phage vB_AbaP_PD-AB9) |
| ORF036 | 22064..23620 | 518 | 99.81% | head-to-tail connector protein  (*Acinetobacter* phage WCHABP5) |
| ORF037 | 23629..24489 | 286 | 100% | capsid assembly scaffolding protein  (*Acinetobacter* phage Abp1) |
| ORF038 | 24505..25536 | 343 | 99.71% | major capsid protein  (*Acinetobacter* phage APK37.1) |
| ORF039 | 25590..25775 | 61 | 98.36% | hypothetical protein  (*Acinetobacter* phage MRABP9) |
| ORF040 | 25931..26491 | 186 | 100% | non-contractile tail tubular protein (*Acinetobacter* phage AbKT21phiIII) |
| ORF041 | 26500..28791 | 763 | 99.87% | non-contractile tail tubular protein (*Acinetobacter* phage vB_AbaP_APK81) |
| ORF042 | 28791..29465 | 224 | 99.55% | internal virion protein A  (*Acinetobacter* phage vB_AbaP_APK81) |
| ORF043 | 29478..32363 | 961 | 98.44% | internal virion protein B  (*Acinetobacter* phage vB_AbaP_APK2-2) |
| ORF044 | 32373..35471 | 1032 | 99.13% | peptidoglycan lytic exotransglycosylase (*Acinetobacter* phage vB_AbaP_APK37) |
| ORF045 | 35477..37558 | 693 | 99.13% | non-contractile tail fiber protein (*Acinetobacter* phage vB_AbaP_ABWU2101) |
| ORF046 | 37572..37907 | 111 | 99.10% | holin  (*Acinetobacter* phage SH-Ab 15519) |
| ORF047 | 37894..38451 | 185 | 98.38% | endolysin  (*Acinetobacter* phage AB_NC12) |
| ORF048 | 38511..38819 | 102 | 100% | terminase small subunit  (*Acinetobacter* phage Abp1) |
| ORF049 | 38829..40766 | 645 | 99.69% | terminase large subunit  (*Acinetobacter* phage YZ2) |
| ORF050 | 40763..40900 | 45 | 97.78% | DNA-binding protein  (*Acinetobacter* phage vB_AbaP_B3) |
| ORF051 | 40857..41060 | 67 | 100% | hypothetical protein  (*Acinetobacter* phage phiAB1) |
| ORF052 | 41071..41253 | 60 | 100% | hypothetical protein  (*Acinetobacter* phage SH-Ab 15519) |
| ORF053 | 41380..41637 | 85 | 80.95% | hypothetical protein  (*Acinetobacter* virus vB_AbaP_AGC01) |

*No significant similarities were found.
