# Supplementary material for: Characterization of phage AbpL with a terminally redundant genome and its therapeutic potential against drug-resistant Acinetobacter baumannii infections
Source: Front Cell Infect Microbiol. 2026 Feb 3;16:1760018. doi: 10.3389/fcimb.2026.1760018 (PMC12960631; doi:10.3389/fcimb.2026.1760018)
Supplement: Supplementary file 1 [file DataSheet1.pdf]

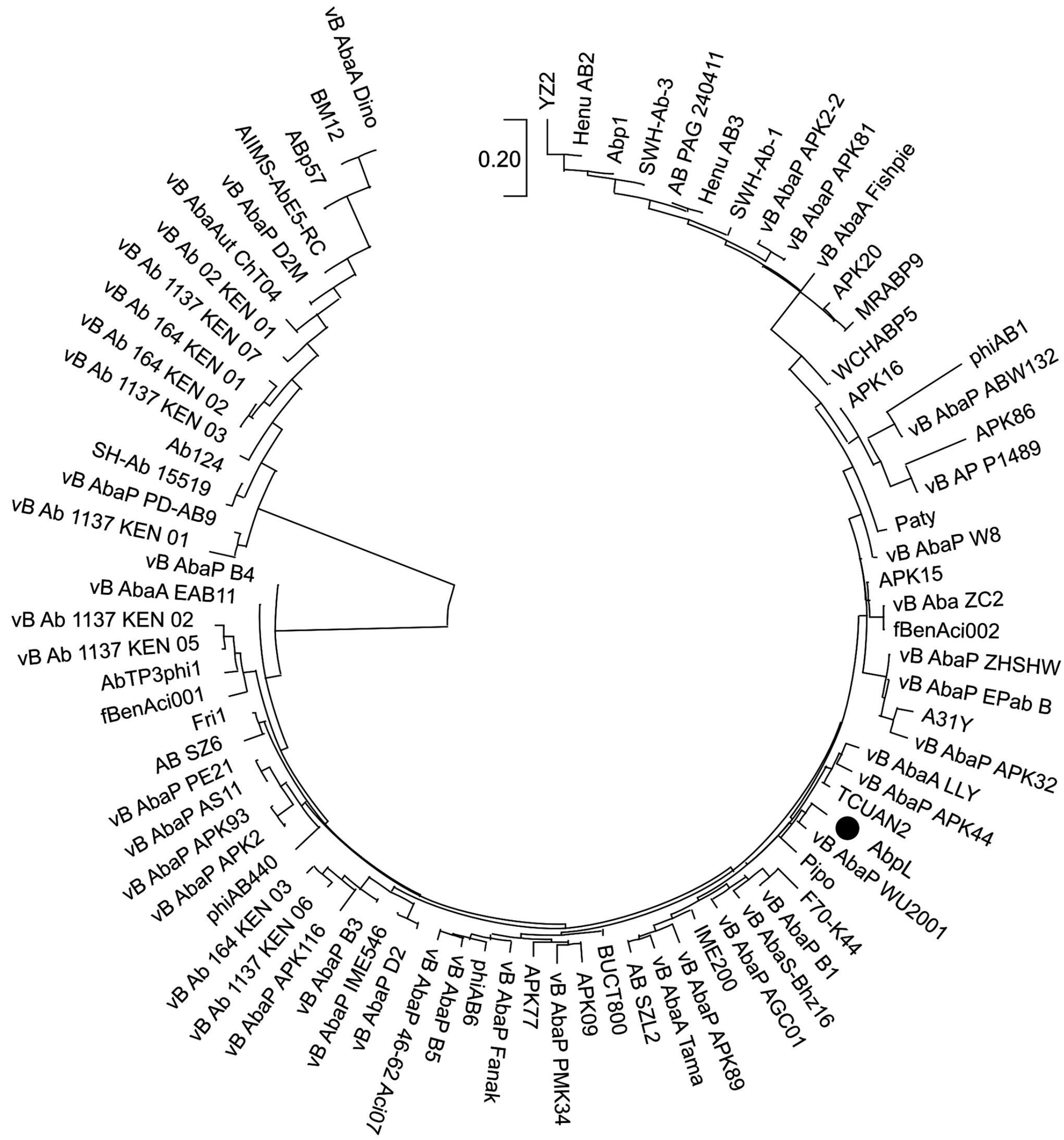

Figure S1. Phylogenetic analysis of the terminal repeat sequence in phage AbpL. A total of 83 corresponding sequences from *A. baumannii* phages were included in the analysis. Scale bar: relative genetic distance.
